# Comparison of meiotic transcriptomes of three maize inbreds with different origins reveals differences in cell cycle and recombination

**DOI:** 10.1186/s12864-022-08922-w

**Published:** 2022-10-12

**Authors:** Nelson Garcia, Lu Yin, Stefanie Dukowic-Schulze, Claire Milsted, Penny M. A. Kianian, Shahryar Kianian, Wojciech P. Pawlowski, Changbin Chen

**Affiliations:** 1 Department of Horticultural Science, University of Minnesota, Saint Paul, MN USA; 2 Present Address: Sound Agriculture, 5858 Horton St, Emeryville, CA USA; 3 School of Life Sciences, Arizona State University, Tempe, AZ USA; 4 Microvascular Biology and Pathobiology, University of Heidelberg, Mannheim, Germany; 5 PepsiCo Inc., 210 Borlaug Hall, 1991 Upper Buford Circle, Saint Paul, MN USA; 6 Department of Agriculture – Agricultural Research Service, Cereal Disease Lab, U.S., Saint Paul, MN USA; 7 School of Integrative Plant Science, Cornell University, Ithaca, NY USA

**Keywords:** Double strand break (DSB), Crossover (CO), Recombination, Meiosis, Gene expression patterns, Meiocytes, Maize

## Abstract

**Background:**

Cellular events during meiosis can differ between inbred lines in maize. Substantial differences in the average numbers of chiasmata and double-strand breaks (DSBs) per meiotic cell have been documented among diverse inbred lines of maize: CML228, a tropical maize inbred line, B73 and Mo17, temperate maize lines. To determine if gene expression might explain these observed differences, an RNA-Seq experiment was performed on CML228 male meiocytes which was compared to B73 and Mo17 male meiocytes, where plants were grown in the same controlled environment.

**Results:**

We found that a few DSB-repair/meiotic genes which promote class I crossovers (COs) and the *Zyp1* gene which limits newly formed class I COs were up-regulated, whereas *Mus81 homolog 2* which promotes class II COs was down-regulated in CML228. Although we did not find enriched gene ontology (GO) categories directly related to meiosis, we found that GO categories in membrane, localization, proteolysis, energy processes were up-regulated in CML228, while chromatin remodeling, epigenetic regulation, and cell cycle related processes including meiosis related cell cycle processes were down-regulated in CML228. The degree of similarity in expression patterns between the three maize lines reflect their genetic relatedness: B73 and Mo17 had similar meiotic expressions and CML228 had a more distinct expression profile.

**Conclusions:**

We found that meiotic related genes were mostly conserved among the three maize inbreds except for a few DSB-repair/meiotic genes. The findings that the molecular players in limiting class I CO formation (once CO assurance is achieved) were up-regulated and those involved in promoting class II CO formation were down-regulated in CML228 agree with the lower chiasmata number observed in CML228 previously. In addition, epigenetics such as chromatin remodeling and histone modification might play a role. Transport and energy-related processes was up-regulated and *Cyclin13* was down-regulated in CML228. The direction of gene expression of these processes agree with that previously found in meiotic tissues compared with vegetative tissues. In summary, we used different natural maize inbred lines from different climatic conditions and have shown their differences in expression landscape in male meiocytes.

**Supplementary Information:**

The online version contains supplementary material available at 10.1186/s12864-022-08922-w.

## Background

Meiosis is essential for sexually reproductive eukaryotes. During meiosis, homologous chromosomes pair, recombine and segregate, resulting in haploid gametes after two rounds of cell divisions following one round of genome duplication. Homologous recombination refers to the process of homologous chromosome segments exchange, also known as crossovers (COs). At least one CO per chromosome pair is needed for proper chromosome segregation, whereas no more than four COs per pair chromosomes are commonly observed in most eukaryotes [1]. Meiotic homologous recombination not only facilitates successful reproduction, but also gives rise to novel allelic combinations which are the basis of plant breeding.

At the beginning of prophase I, recombination is initiated by formation of double-stranded-breaks (DSBs) through the enzyme SPO11. The repair of DSBs can lead to CO formation but the majority of DSBs do not result in COs. For example in maize, there can be up to 500 DSBs per meiosis while there are fewer than 20 resulting COs. DSBs are distributed in all chromosome regions including regions lacking COs such as centromeric and pericentromeric regions, whereas CO rates were higher in distal chromosome regions [2, 3]. DSB hotspot sites are generally associated with open chromatin, marked by low nucleosome occupancy or trimethylation of lysine 4 of the H3 histone (H3K4me3), and low levels of DNA methylation [2]. But open chromatin by itself may not be sufficient to predict a DSB hotspot [2]. Within genes, ~ 30% of COs are located within 2 kb upstream from transcription start sites and ~ 20% COs are located within 2 kb downstream of transcription termination sites [3].

The MRN (MRE11, RAD50, NBS1) complex creates single-strand overhangs at the ends of the DSB, resulting in single-end invasion which is loaded by two bacterial recombinase RecA-like proteins, RAD51 and DMC1 [4–7]. Ataxia telangiectasia mutated- and RAD3-related (ATR) and breast cancer protein (BRCA2) regulates DMC1 loading and RAD51 activity, respectively. The single-end invasion intermediates, through further DNA synthesis, ligation, and second-end capture, form recombinant intermediates called double Holliday junctions (dHJs) [7], which can be resolved into the class I COs. Class I COs are affected by interference, whereas class II COs are not affected by interference. Class II COs account for ~ 15% of COs in maize [8] and only MUS81 has been characterized as required to resolve dHJs (and RAD1/XPF1/MEI-9 has a potential role) [6, 9]. For the class I CO pathway, the major players MSH4 and MSH5 form a heterodimer that likely stabilizes the intermediates that lead to dHJs [7], where MER3, HOC1 (ZIP2), HEI10, MLH1 and MLH3 are also involved [6].

Starting in mid-prophase I, a tripartite structure call synaptonemal complex forms between two homologous chromosome axes which is also important for meiotic recombination [6]. Lateral element components ASY1 and AFD1, and central element component ZYP1 are essential players in chromosome pairing [10]. ZYP1, which is compared to the teeth of the molecular zipper that is in the synaptonemal complex, plays a role both in ensuring one CO per pair of chromosomes (CO assurance) and in limiting closely spaced CO formation once that goal is reached [6, 11].

In addition to homologous recombination, there are other DNA damage repair mechanisms including non-homologous end-joining, base excision repair, nucleotide-excision repair, and mismatch repair [12]. Environmental stresses and various exogeneous factors such as natural UV radiation and genotoxic compounds can also cause DNA breaks and damage [13]. When DNA damage is not too severe, cell cycle checkpoints are activated to transiently inhibit cell proliferation and a series of DNA repair pathways are activated. This process usually requires one of the two phosphatidylinositol-3-OH kinase-like kinases: ataxia telangiectasia mutated (ATM) and ATR [14]. ATM is typically activated by DSBs, while ATR is usually activated by stalled DNA replication fork [14]. ATM and ATR are involved in activation of WEE1 kinase which operates as an off switch for the cyclin-dependent kinase (CDK) activity in plants which, together with a regulatory cyclin subunit, is required for cell cycle progression [14].

Interestingly, the maize inbred line CML228 has fewer DSBs and chiasmata (cytological manifestations of COs) than other lines such as B73 and Mo17, studied under a controlled environment [15]. B73 is the first reference genome in maize [16] and together with Mo17 is one of commonly used model lines in maize genetic studies, adapted to temperate climate [17, 18]. CML228 was initially developed from Suwan-1, which is adapted to a low-land tropical climate where high temperature and high light are typical [19]. This study by Sidhu et al. [15] raises several questions: why does CML228 have fewer chiasmata and what contributes to this difference in CO number among different lines? Could it be due to differences in adaptation to different environments? What genes are differentially expressed among CML228 and the other lines, particularly those related to DSB-induced repair?

Our ability to investigate transcriptomes of different lines specifically within male meiocytes eliminates confounding expression from other tissues. Dukowic-Schulze, Harris, et al. [20] found some significant differences between expression profiles of meiocytes versus seedlings. Here we present the use of meiocytes of maize lines of different origins to study their transcriptome expression profiles. Using RNA-seq from male meiocytes of the tropical maize inbred line CML228 with a lower chiasmata number and the temperate maize lines B73 and Mo17 grown under a greenhouse at University of Minnesota, St. Paul, we compared their gene expression profiles, particularly in relation to meiotic homologous recombination.

## Results

**Fig. 1:**
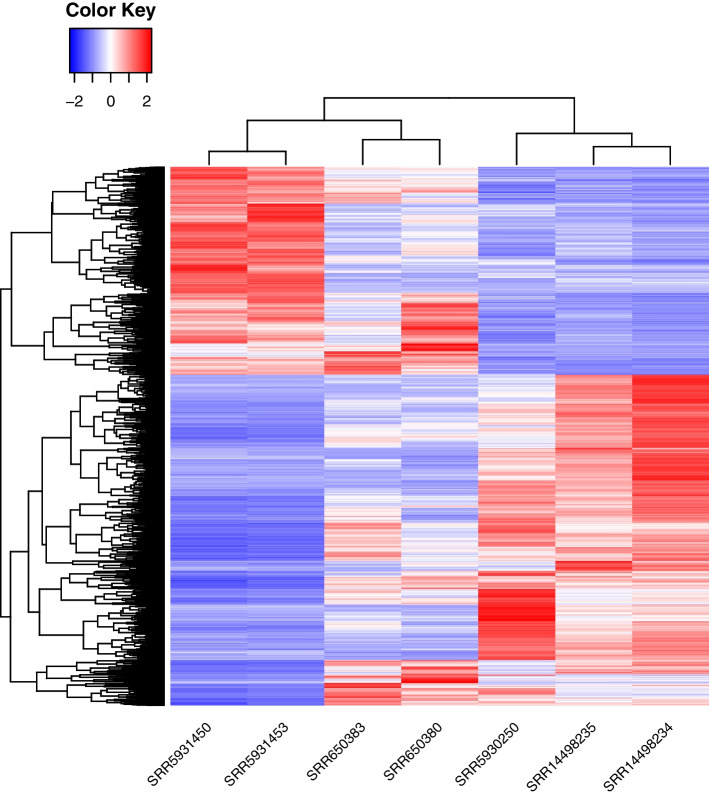
Z-scores showing expression profiles of the 3,664 differentially expressed genes of maize inbred lines B73 (SRR650383 and SRR650380), Mo17 (SRR5931453 and SRR5931450), and CML228 (SRR5930250, SRR14498235, and SRR14498234) meiocytes

We found 3,664 genes differentially expressed among the male meiocytes of the three maize inbred lines (Fig. 1). About 37 to 70 million raw reads of B73 meiocytes were aligned to the B73 v5 reference genome, 13 to 17 million reads of Mo17 meiocytes were aligned to Mo17 v1 reference genome, and 38 to 41 million reads of CML228 meiocytes were aligned to the CML228 v1 reference genome [21, 22] (Supplementary Table 1). Among the genes that had reads per kilobase per million (RPKM) values greater than 2, a total of 14,206 genes were annotated in all three reference genomes (Supplementary Fig. 1). Out of these, 3,664 genes showed a false discovery rate (FDR) < 0.01 and a fold change greater than 2 among B73, Mo17 and CML228 (Supplementary Table 2). The replicates of the expression profiles of the 14,206 genes and the 3,664 genes of each line clustered closer together (Supplementary Fig. 2; Fig. 1). Within the 3,664 differentially expressed genes, there were 2,243 genes up-regulated in CML228 compared to B73 or Mo17 and 1,421 genes down-regulated in CML228 (Fig. 1).

When aligning all B73, Mo17, and CML228 meiocyte transcriptomes to the same reference (the B73 v5 reference, the Mo17 v1 reference, or the CML228 v1 reference), the expression profiles of Mo17 replicates again clustered closer with B73 replicates than CML228 replicates (Supplementary Fig. 3). The finding that alignment of the transcriptomes to the same reference genome show similar expression patterns as alignment to their corresponding reference genome (Fig. 1) confirms that the differential gene expression found was not due to differences in the reference genome but indeed due to the expression of these genes themselves. We will focus on the results obtained by aligning the transcriptomes of each meiocyte to their corresponding reference genome.

**Fig. 2:**
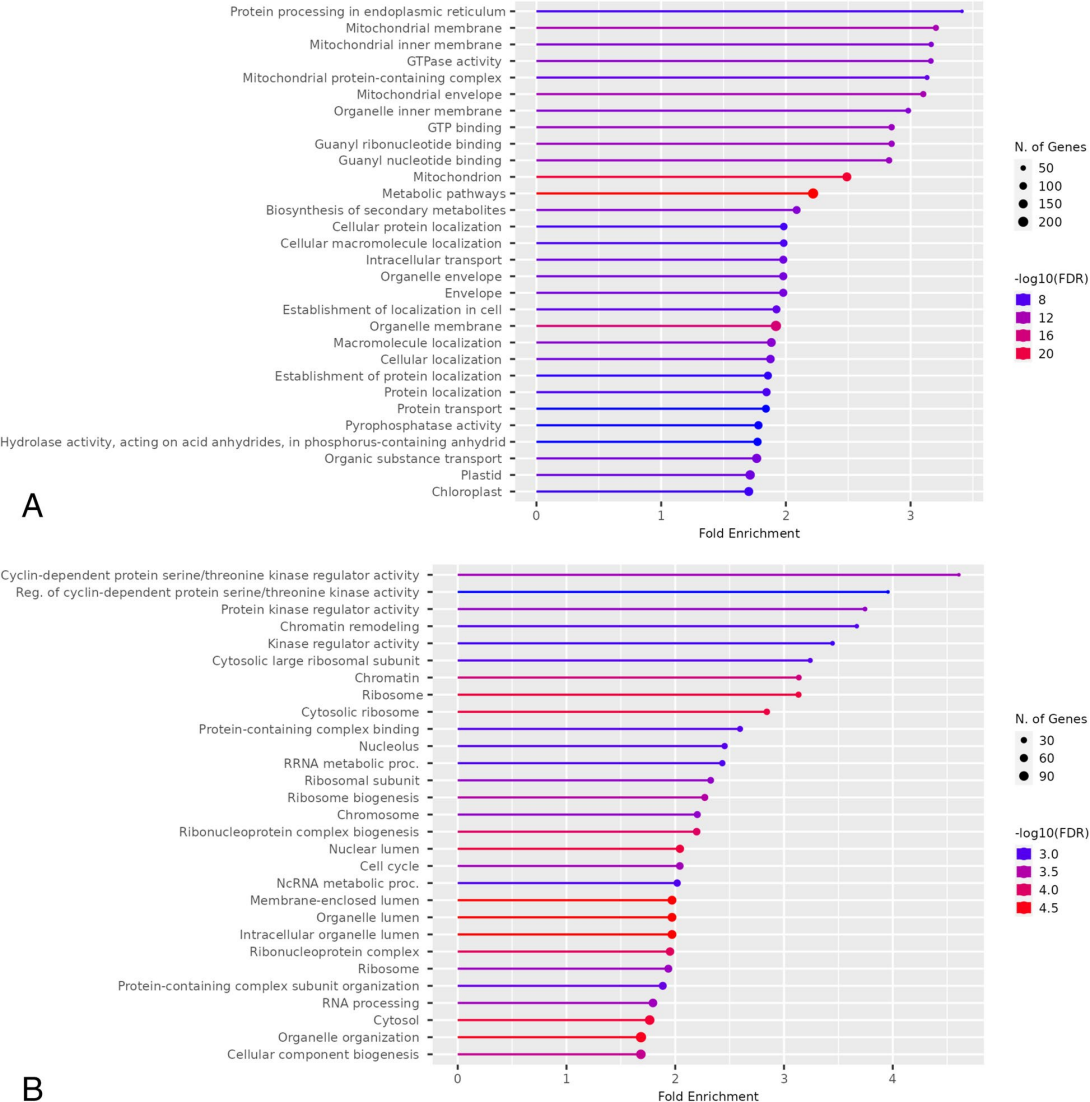
Top 30 Gene Ontology (GO) terms associated with **(A)** the 2,243 up-regulated genes and **(B)** the 1,421 down-regulated genes in CML228 meiocytes compared to B73, Mo17 meiocytes

**Fig. 3:**
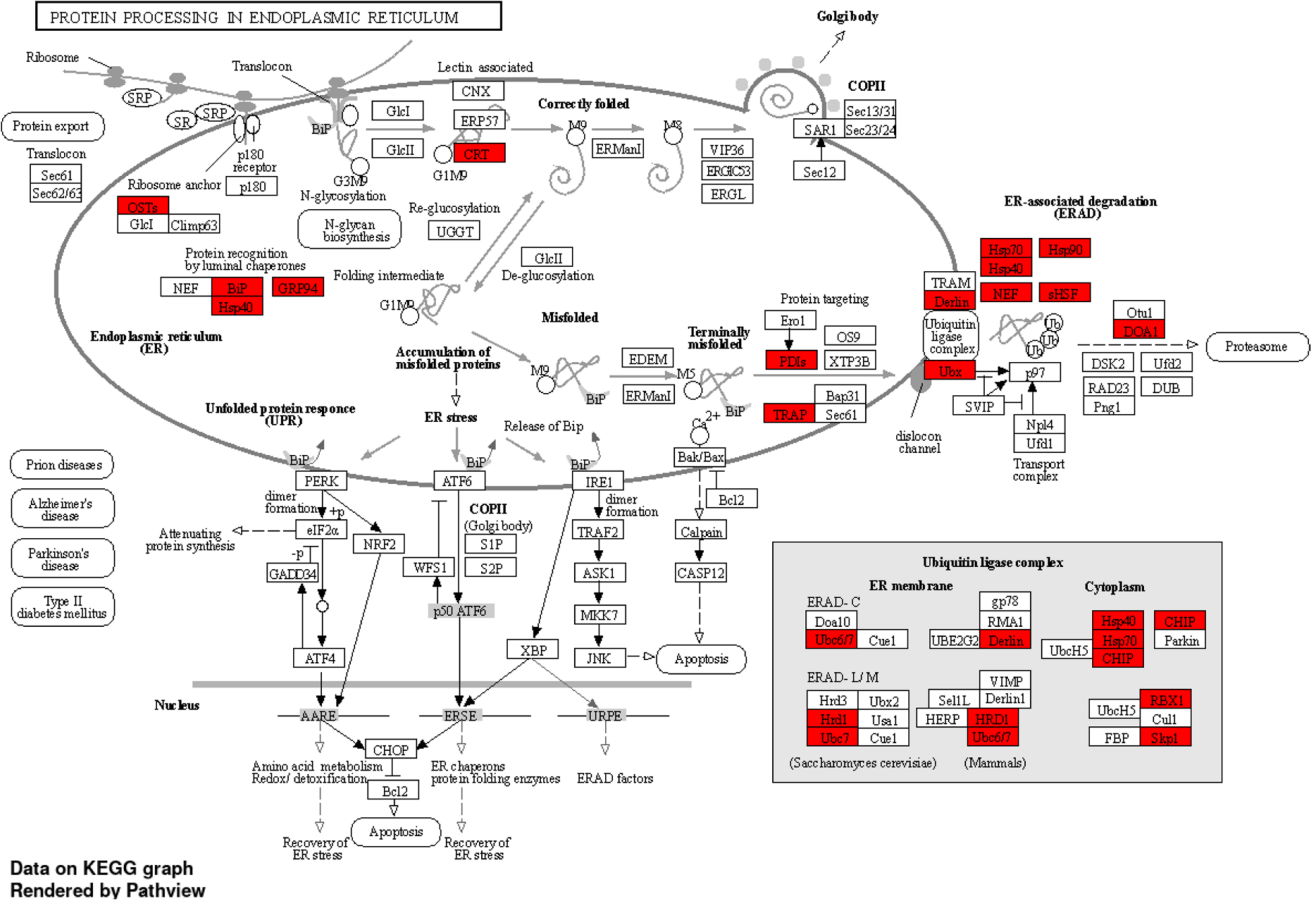
One example of the top KEGG pathway associated with up-regulated genes in CML228 compared with B73 and Mo17 [23–27]

The enriched gene ontology (GO) categories in the 2,243 up-regulated genes in CML228 include membrane, protein/cellular localization, and transport (Fig. 2A) and that in the 1,421 down-regulated genes in CML228 include cell cycle/cyclin kinase genes, ribosome/RNA processing, chromatin remodeling/chromosome, and organelle lumen/nucleolus (Fig. 2B). Kyoto Encyclopedia of Genes and Genomes (KEGG) pathways associated with up-regulated genes in CML228 include protein processing in endoplasmic reticulum (ER), biosynthesis of cofactors and amino acids, metabolism of carbon, cysteine, methionine, and pyruvate, glycolysis, secondary metabolites, and endocytosis (Fig. 3); KEGG pathways associated with down-regulated genes in CML228 include ribosome, starch and sucrose metabolism, and circadian rhythm. Membrane genes, protein/cellular localization, and transport genes were repeatedly detected to be enriched in up-regulated genes in CML228 when being aligned to the same B73 v5 reference genome, Mo17 v1 reference genome, or CML228 v1 reference genome (Supplementary Figs. 4A, 5A, and 6A). Similarly, ribosome, and chromatin genes were repeatedly detected to be enriched in down-regulated genes in CML228 when being aligned to the same B73 v5 reference genome, Mo17 v1 reference genome, or CML228 v1 reference genome (Supplementary Figs. 4B, 5B, and 6B).

### Selected DNA damage repair genes were differentially expressed among the tested inbred lines

We examined several well-characterized meiotic DNA damage repair genes and found that some of them were differentially expressed in CML228, B73 and Mo17 (Table 1), while other genes were non-differentially expressed (Supplementary Table 3). Some of the players involved in early steps of meiosis and single-end invasion such as *Dmc1, Rad51d, Rad51e*, and *Brca2* were up-regulated in CML228 meiotic transcriptome compared with that of B73 and Mo17 (Table 1; Supplementary Table 3). *Zyp1* and *Zip4* which play a role in limiting class I COs were up-regulated in CML228. *Mer3* and *Mlh1* (a log_2_ fold change of -1.02, FDR = 0.016) which promote class I COs were up-regulated in CML228. *Mus81 homolog 2* (a log_2_ fold change of 0.97, FDR = 0.006) which promotes class II COs was down-regulated in CML228. *Atr*, regulating *Dmc1* loading, was down-regulated in CML228 (Table 1; Supplementary Table 3).

**Table 1:** Selected significantly differentially expressed DNA damage repair genes: gene function and gene expression levels (shown as Z-scores) among the male meiocytes of temperate maize lines B73 and Mo17 and the tropical maize line CML228

Gene name	Gene ID	Enriched in GO analysis	CML228			Mo17		B73		Gene function
			SRR5930250	SRR14498235	SRR14498234	SRR5931453	SRR5931450	SRR650380	SRR650383	
*Zyp1*	Zm00001eb423930	-	1.40	0.86	0.89	-0.79	-0.88	-0.81	-0.67	Chromosome synapsis
*Dmc1*	Zm00001eb163340	-	0.10	0.61	1.44	-1.13	-1.16	-0.61	0.74	Homology search
*Mer3*	Zm00001eb184840	-	0.98	0.15	0.91	-1.32	-1.35	-0.15	0.77	Meiosis-specific DNA helicase
*Zip4*	Zm00001eb145140	Up	-0.29	1.13	1.71	-0.81	-0.75	-0.59	-0.39	Chromosome synapsis
*Rad51d*	Zm00001eb329200	-	-0.58	0.39	0.88	-1.10	-1.36	0.62	1.13	Homology search
*Rad51e*	Zm00001eb355220	-	0.73	0.09	1.29	-1.35	-1.19	-0.26	0.69	Homology search
*Brca2*	Zm00001eb419130	-	1.67	0.35	0.43	-1.29	-0.94	-0.53	0.30	Homology search
*Tti2*	Zm00001eb226270	-	1.24	0.33	0.91	-1.12	-1.42	-0.31	0.36	Stabilization of phosphatidylinositol 3-kinase-related kinases
Mlh1*	Zm00001eb362590	Up	1.19	-0.11	0.76	-0.92	-1.36	-0.55	1.00	Interference crossover formation
*Mis12b*	Zm00001eb065850	Up	0.29	0.98	1.64	-0.89	-0.93	-0.62	-0.46	Kinetochore protein
*e2f12*	Zm00001eb280090	-	0.46	-0.19	-0.22	-1.05	-1.14	0.35	1.78	Suppression of endocycle
*e2f13*	Zm00001eb194770	-	0.50	0.76	1.69	-0.85	-0.88	-0.83	-0.39	Suppression of endocycle
*Atr1*	Zm00001eb228310	Down	-1.12	-0.93	-0.63	1.09	1.44	-0.24	0.40	Regulating *Dmc1* loading
Phr1*	Zm00001eb015750	-	-0.39	-0.96	-1.30	0.92	1.01	-0.39	1.12	DNA repair
Mus81 homolog2*	Zm00001eb141330	-	-0.27	-1.24	-0.94	1.07	1.41	-0.45	0.43	Noninterference crossover junction endonuclease
*Mpk3*	Zm00001eb013100	Down	-0.56	-0.71	-0.77	1.30	1.58	-0.25	-0.59	Cell cycle progression upon UVB stress

^*^ not differentially expressed, but nearly significant

Out of the DNA repair genes up-regulated in CML228 (Table 1; Supplementary Table 3), the expression of *Zyp1* was validated through real-time PCR (qPCR). The qPCR results showed that although not significant, the relative expression of *Zyp1* was numerically higher in CML228 compared with B73 or Mo17 using ANOVA and Tukey’s HSD test (Supplementary Fig. 7).

Looking at the GO analysis, no meiosis or recombination categories were found (Fig. 2) but when the meiotic transcriptomes were aligned to the same reference genome, regulation of DNA recombination was found to be down-regulated in CML228 (Supplementary Figs. 4B, 5B, and 6B). Similarly, no KEGG pathway enrichment analysis showed any direct relations to DNA repair or meiosis.

Yet GO terms such as chromatin, chromosome, chromatin remodeling, nucleolus, ribosome, non-coding RNA (ncRNA) metabolic processing/RNA processing were enriched in down-regulated genes in CML228 (Fig. 2B). There were 40 genes in the “chromosome” GO category, 25 genes in the “chromatin” GO category, and 16 genes in “chromatin remodeling” category (Table 2). There were also 327 ribosome related genes and 145 ribosomal RNA/non-coding RNA (ncRNA)/RNA processing genes. Consistently, KEGG pathway analysis also showed that genes involved in ribosome were down-regulated in CML228. Chromatin-related genes include histone genes, high mobility group (HMG) genes, transcription factors, nucleosome/chromatin assembly genes, and cell cycle/circadian rhythm related genes (Table 2). One example of the genes was *Chromatin complex subunit A 101* (*Chr101*), also known as *Ddm1*. Two E2f genes were detected, with a role in transcriptional regulation of DNA damage response in plants (Table 1).

**Table 2:** Significantly down-regulated genes (FDR < 0.01) in the tropical maize line CML228 enriched in chromatin, chromosome, and chromatin remodeling gene ontology categories: log_2_ fold change and gene expression levels (shown as Z-scores) among the male meiocytes of temperate maize lines B73 and Mo17 and CML228

Gene name	Gene ID	Log_2_(FC)	CML228			Mo17		B73	
			SRR5930250	SRR14498235	SRR14498234	SRR5931453	SRR5931450	SRR650380	SRR650383
Cl4586_1A, KID-containing protein	Zm00001eb035860	1.27	-0.92	-0.62	-1.08	1.69	0.32	0.78	-0.17
Retinoblastoma family 3	Zm00001eb037120	1.14	-0.48	-0.24	-0.68	1.40	1.47	-0.85	-0.63
High mobility group (HMG) family A 102	Zm00001eb041850	1.84	-1.17	-0.55	-0.76	1.26	1.10	0.75	-0.63
NA	Zm00001eb060330	1.61	-0.80	-0.50	-0.59	1.46	1.45	-0.57	-0.45
Pco124429	Zm00001eb065860	1.28	-1.38	-0.43	-0.63	1.19	1.42	0.10	-0.26
Histone 1(H1) 101	Zm00001eb073400	1.17	-1.40	-0.59	-0.73	1.58	0.74	0.33	0.06
NA	Zm00001eb103320	1.49	-1.09	-0.82	-1.05	0.88	1.37	0.12	0.59
Cl44900_1(183), Sld5	Zm00001eb108600	1.84	-0.74	-1.12	-1.01	0.84	0.90	-0.14	1.27
NA	Zm00001eb114240	2.46	-0.88	-0.79	-0.98	1.18	1.49	-0.06	0.03
IDP583	Zm00001eb146760	4.03	-0.77	-0.73	-0.71	1.34	1.54	-0.35	-0.32
NA	Zm00001eb160010	1.03	-1.17	-0.57	-1.13	1.02	1.28	0.59	-0.02
NA	Zm00001eb184560	1.12	-0.86	-0.39	-0.56	1.53	1.34	-0.32	-0.74
NA	Zm00001eb215110	2.03	-0.98	-0.52	-0.84	0.98	1.69	0.17	-0.50
NA	Zm00001eb229690	1.26	-0.76	-1.05	-0.78	1.03	1.49	-0.43	0.51
Si606066c12	Zm00001eb266890	3.21	-0.69	-0.57	-0.57	1.36	1.56	-0.57	-0.53
Profilin homolog4	Zm00001eb270920	1.59	-0.85	-0.30	-0.12	1.50	1.32	-0.82	-0.73
HMG-transcription factor 12	Zm00001eb282920	1.34	-1.09	-1.02	-0.59	0.29	0.25	0.38	1.77
NA	Zm00001eb302390	6.82	-0.90	-0.90	-0.90	0.55	0.01	0.35	1.78
Cl57132_1	Zm00001eb379890	1.57	-1.01	-0.63	-1.10	1.01	1.37	-0.29	0.65
Precocious dissociation of sisters-like 5 (Pds5)	Zm00001eb380840	1.53	-0.92	-0.49	-0.94	1.50	1.29	-0.34	-0.10
NA	Zm00001eb421110	1.03	-0.54	-0.67	-0.55	0.57	2.06	-0.48	-0.39
Histone 2B3	Zm00001eb099910	1.67	-0.97	-0.93	-1.07	0.55	0.14	0.88	1.40
NA	Zm00001eb110350	1.00	-1.12	-0.34	-1.01	1.50	1.11	0.18	-0.31
Chromatin complex subunit A 101	Zm00001eb117870	1.88	-0.73	-0.61	-0.53	1.70	1.16	-0.69	-0.30
Histone 2B2	Zm00001eb177270	1.03	-1.04	-0.85	-1.25	1.14	0.59	0.66	0.75
HMG-Y-related protein A (Umc1511)	Zm00001eb179440	1.68	-1.04	-0.63	-0.88	1.02	1.48	0.52	-0.47
NA	Zm00001eb195120	1.28	-0.99	-1.12	-0.93	0.81	0.66	0.27	1.31
Global transcription factor C (Spt16; Gtc102)	Zm00001eb229990	1.21	-0.41	-0.54	-1.07	1.76	1.01	-0.35	-0.39
NA	Zm00001eb231050	1.10	-0.97	-0.71	-0.64	0.60	1.92	0.10	-0.31
Nucleosome/chromatin assembly factor A104	Zm00001eb244920	1.01	0.01	-0.84	-1.00	0.95	1.73	-0.52	-0.32
Global transcription factor A (Spt6; Gtb101)	Zm00001eb291590	1.35	-0.91	-0.46	-1.07	1.38	1.35	-0.32	0.03
Histone 1a	Zm00001eb301680	1.15	-1.11	-0.45	-0.81	1.36	1.36	0.10	-0.44
NA	Zm00001eb304780	1.32	-0.41	-1.03	-1.08	1.18	1.44	-0.30	0.20
Histone H2b (Umc1268)	Zm00001eb368020	1.23	-0.16	-1.14	-1.21	1.19	1.31	0.18	-0.18
Cryptochrome3	Zm00001eb382070	2.22	-1.13	-0.97	-1.02	0.99	0.50	0.54	1.10

### Up-regulated membrane-related genes and down-regulated cell cycle genes in CML228

While the three inbred lines had distinct expression profiles, the expression profiles of the differentially expressed genes of B73 (SRR650380 and SRR650383) and Mo17 (SRR5931450 and SRR5931453) were closer to each other than to CML228 (SRR5930250, SRR14498235, and SRR14498234) (Fig. 1). Meiocytes of Mo17 and B73 also clustered closer together than that of CML228 for the 14,206 genes in sample-to-sample distance matrix and in principal component 1 (Supplementary Fig. 2).

GO enrichment analysis showed an up-regulation of genes involved in membrane and protein localization in the tropical maize line CML228. In addition, hydrolase and pyrophosphatatse genes were up-regulated in CML228 (Fig. 2A). Consistently, KEGG pathway enrichment analysis also revealed an up-regulation of protein processing in ER degradation genes in CML228 such as the chaperone (BiP), the protein disulfide isomerase (PDI), and several heat shock proteins (HSPs) (Fig. 3). Genes involved in energy production were also up-regulated in CML228, including guanosine triphosphate (GTP) binding, GTPase activity, guanyl ribonucleotide binding, and mitochondrion (Fig. 2A). KEGG pathway analysis also showed that genes in carbon metabolism were up-regulated in CML228.

Cell cycle genes were down-regulated in CML228. A total of 51 genes were in the “cell cycle” GO category, 14 of which were also in the “cyclin-dependent serine/threonine related kinases” GO categories (Fig. 2B; Supplementary Table 4). For the expression levels of these genes, reported as Z-scores, all 51 were lower in CML228 compared to Mo17 and 18 were lower in CML228 compared to B73 (Supplementary Table 4). Particularly, we detected seven cyclin genes*,* four *cyclin-dependent kinase inhibitor (Cki1)* genes, one *Cdk* gene, *Mitogen-activated protein (MAP) kinase* 3 (*Mpk3),* and *Retinoblastoma family 3* gene (Supplementary Table 4). Additionally, genes involved in microtubule structure and cell plate such as *Precocious dissociation of sisters-like 5* (*Pds5*) and protein TPX2 were down-regulated in CML228 (Supplementary Table 4). Genes in the circadian rhythm KEGG pathway were also down-regulated in CML228.

To look into these cell cycle genes further, KEGG analysis of these 51 cell cycle genes revealed enrichment of DNA replication, mismatch repair and nucleotide excision repair pathways (Supplementary Fig. 8). Particularly, replication protein A (RPA) and replication factor C (RFC) were repeatedly detected in these pathways (Supplementary Fig. 8). In addition, *Atr, Wee1*, *Rad1*, *protein partner of SLD five 1* (*Psf1*) and the helicase mini-chromosome maintenance 2 (MCM2) were detected (Supplementary Table 4, Supplementary Fig. 8A).

## Discussion

We were particularly interested in understanding molecular players involved in DSB-induced repair or meiosis, so we first examined several key genes involved in such pathways and whether they were differentially expressed in the three maize inbreds that were previously shown with different chiasmata and DSB numbers. Except for a few meiotic DNA repair genes, the expression of many meiotic DNA repair genes were conserved across the three inbreds. Some of the key molecular players involved in early steps of meiosis and class I CO formation as well as limiting class I CO formation were up-regulated in CML228, whereas *Mus81 homolog 2* promoting class II CO formation was down-regulated in CML228. It could be that once one CO is formed per pair of chromosomes, both class I and II COs were inhibited in CML228 compared with B73 and Mo17. The finding that expression profiles of B73 and Mo17 being closer to each other than to CML228 is consistent with the fact that both B73 and Mo17 are temperate maize lines while CML228 is a tropical maize line. Particularly, transport and energy related processes were up-regulated in CML228, while chromatin, nucleolus, RNA processing, and growth-related processes such as cell cycle were down-regulated in CML228.

### Most DNA repair genes except a few were conserved among the three inbreds

The finding that we did not detect GO categories or KEGG pathways directly involved in DNA damage repair or meiosis suggests that the expression of these genes was mostly conserved across the three maize inbreds examined. The up-regulation of a few genes involved in early DSB formation and single-end invasion in CML228 including *Dmc1, Rad51d, Rad51e* and *Brca2* could be due to the differences in cell cycle progression which proceeds the meiotic cycle. The result on the up-regulation of both the class I CO-promoting *Mer3* and *Mlh1* and class I CO limiting *Zyp1* in CML228 has several explanations. *Mlh1* acts downstream of *Msh5* in the class I CO pathway and *Mer3* is a meiosis-species helicase that process DSBs into dHJs [28], both of which might or might not lead to class I COs. It could also be that once the one CO per pair of chromosomes was formed, *Zyp1* acted to limit other close-by formation of COs. In Arabidopsis, such roles of *Zyp1* was found [11]. The *zyp1* mutant has defects in synaptonemal complex formation, resulting in more class I COs that are more distantly spaced [11]. *Zip4,* which was also up-regulated in CML228, assists *Zyp1* to polymerase along the chromosomes [29]. *Mus81 homolog 2* that was down-regulated in CML228 is a major player in the noninterfering CO pathway [6, 28, 30]. It could be that in CML228 class II COs were lower than B73 and Mo17, and class I COs could be lower once the CO assurance is achieved, which resulted in an overall lower chiasmata as observed in CML228 [15]. Future studies should be done using mutants in *Mus81* or a double-mutant in *Zyp1* and *Zip4* in the CML228 background to confirm this. Since there is a homolog 2 of *Mus81* in maize, perhaps a double mutant for this gene also needs to be considered.

This study was chosen to be conducted in the controlled environment (the greenhouse) as a follow-up to the Sidhu et al. [15] study which found, under controlled environments (the growth chamber), the lower DSB and chiasmata numbers of CML228 compared with the other inbreds. We do not yet know how the three lines would perform under CML228’s native tropical climate or the temperate climate. It could be that under higher temperature, COs are generally inhibited in all maize lines, but perhaps CML228 has adapted some mechanisms that counteract this inhibition on CO number. Arrieta et al. [31]. Perhaps CML228 maize, naturally adapted to crossover-inhibiting heat stress, displays fewer COs when grown in a controlled environment, which could be a different story when all lines are grown under heat stress. Future studies could examine this in comparison with the current study and the study of Sidhu et al. [15]. However, there are challenges of such studies given that meiosis can fail under high heat environments [32].

Chromatin structure and potential epigenetic differences such as ribosome-related genes existed in these maize inbreds. Epigenetic modifications mediate DNA damage and repair through chromatin remodeling, DNA methylation, histone modifications and RNA silencing. Most of these epigenetic modifying genes were down-regulated in CML228. The finding of nucleosome genes down-regulated in CML228 agrees with the literature. Kianian et al. [3] found that nucleosome occupancy patterns at CO sites were different between the crosses B73 × Mo17 and B73 × CML228 in maize. Chromatin remodeler *Ddm1* or *Chr101,* down regulated in CML228, was involved in homology directed repair such as single-strand annealing and homologous recombination at DSB sites by regulating chromatin structure [12]. In maize, the expression of *Chr101* increased as the hybrid B73/Mo17 matured [33]. Genes involved in chromatin modification were also found to be highly expressed in meiocytes compared to seedling tissues [34–36]. In addition, CO distribution along the chromosome could also be different, which was not detected by our approach. For example, barley exposed to moderate heat stress showed a significant decrease in distal COs and an increase in interstitial COs [32]. In all, there is a lack of evidence supporting the role chromatin remodelers/DNA methylation in DNA damage response [12]. Also the roles of major ncRNAs in DNA damage response remain poorly described [12], though certain phased small RNAs were previously found to be enriched in meiocytes [37].

After all, this study only examined the zygotene stage transcriptomes of these maize inbreds. Very large expression changes have been observed in leptotene and zygotene in maize anthers, but the timing of these changes depends on the genetic backgrounds [38, 39]. Both the W23 inbred line and a B73/A1888 hybrid up-regulated a similar set of genes early in meiosis [38, 39]. But the W23 inbred had its major meiotic transcriptome change earlier—in leptotene, whereas the B73/A188 hybrid had the transcriptome change later—in zygotene [38, 39]. It could be that the best stage to capture the major meiotic transcriptome variation in CML228 is not zygotene (e.g. leptotene) as B73 and Mo17. This, however, requires detailed temporal expression profiles of each of these three inbreds in order to determine the optimal sampling time for each line and whether this time differs for each line. In all, consistent with the studies by Nelms and Walbot, the transcriptomes of meiosis related genes are mostly conserved across the maize inbreds.

### Altered expression of genes related to adaptations of the inbreds

First of all, the finding of the meiotic expression profiles of B73 clustered closer together with Mo17 than to CML228 indicates that there was some common ground between B73 and Mo17, very likely the temperate climatic zones they both adapted to. However, we did not directly study the effect of temperature or other environmental stresses on meiotic transcriptomes, so we cannot say anything more than this except that this might be an interesting area for future studies. As our study was examining the meiotic transcriptomes, we mostly focused on the meiosis/DNA repair processes. Here we only briefly talk about the other cellular processes that were different among the inbreds.

A higher rate of protein transport and localization was observed in CML228, which was also observed previously to be enriched in meiocyte transcriptomes compared with seedling transcriptomes [34–36]. Together with finding of the protein processing in ER enriched KEGG pathway in up-regulated genes in CML228, these results suggest that CML228 might utilize proteolysis as a way to adapt to the tropical stressed environments. The chaperone BiP can bind to misfolded proteins to be degraded through the proteasome, PDI is an abundant oxidoreductase present in eukaryotic ER and catalyzes the folding of proteins, whereas HSPs facilitates both correct folding and degradation [40]. Together, these players might help getting rid of the negative consequences in metabolism due to tropical stresses.

A down-regulation of cell cycle genes such as cell plate formation, sister chromatid cohesion, spindle assembly further indicated that cell division associated processes were delayed in CML228. Some examples include: *Cyclin 1* and *23*, homologs to Arabidopsis *Cyclin B1* and *B2* respectively, which are mitosis-promoting factors and the key regulator for G2/M cell cycle progression in eukaryotes [41],*Cyclin 9* and *26*, type-A2 cyclins, which play a role in G1/S to M phase and regulate the activities of CDKs [42, 43]. Not only players directly involved in cell cycle progression were down-regulated in CML228, but also related processes were down-regulated. Some examples are: microtubule-associated proteins; sister chromatid cohesion genes, which play a role in the cohesion of sister chromatids from the completion of S phase until their segregation in anaphase [44]; protein TPX2, which plays a role in pre-spindle assembly during late prophase at the onset of mitosis before nuclear envelope breakdown [45]; genes in the circadian rhythm KEGG pathway; DNA replication related repair genes such as *Atr, Wee1*, *Rad1,* and *Cdk8*.

It is known that under heat and drought stress, reproductive organs are more susceptible than the vegetative parts [42, 46–48]. Previous studies showed that genes needed for mitochondria function were up-regulated in maize meiocytes compared with other tissues like seedlings, suggesting a higher energy need for meiocytes [34–36, 38]. Our finding that mitochondria-related genes were expressed higher in CML228 meiocytes than B73/Mo17 meiocytes suggests that this energy requirement might be even higher in the inbred line CML228. Whether this higher energy need is related to adaptations to heat is unknown.

Moderate heat stress damage often results in slowed cell proliferation and cell growth [49]. The MAP kinase pathway controls cell cycle progression upon UVB stress which operates independently of ATR [14]. An effect of heat stress on the organization of microtubules has previously been observed in perennial ryegrass and sorghum [50]. Cell cycle genes that were down-regulated in CML228 were enriched in the KEGG pathways DNA replication, mismatch repair pathway and nucleotide excision repair. These pathways were all previously found to be inhibited by heat stress [51].

Interestingly, a down-regulation of type-A2 *Cyclin 9* was previously observed in maize ovaries as opposed to leaf meristems, suggesting a meiosis-specific role of this cyclin [42]. *Cyclin 13* is a type D cyclin (Supplementary Table 4), where D2 cyclins were previously found to be down-regulated in maize during mitosis-to-meiosis transition compared with mitosis cells [38]. Additionally, the authors have observed a loss of ribosomal transcript and an increase in transcripts encoding membrane-bound organelles and mitochondria in meiotic cytoplasm, consistent with our findings. Ribosome elimination was said to play a role during meiosis which could be a mechanism to remove mRNAs and proteins before the gamete stage [38]. Consistently, we’ve found a down-regulation of ribosome related pathways in CML228. We speculate that overall cell division related processes were inhibited in CML228 due to its adaptation to tropical stress, while this consequence was even more pronounced in the meiocytes.

## Limitations and future directions

Some of our discussion was based on the assumption that the differences in the three maize inbreds were due to their adaptations to different climatic zones. While this can be true, there could also be inherit genetic differences among the three lines. But this would be a challenge to study any natural inbred lines. One solution is to create traditional near-isogenic lines through many generations of backcrossing while selected for different alleles at the target gene(s), once the target gene is confirmed.

As expected, the percentage of reads aligned was the highest when the meiocyte transcriptome of each inbred line was aligned to its corresponding reference genome (B73: 86–92%, Mo17: 89–92%), except for CML228 which had the fewest reads aligned to the CML228 v1 reference (CML228: 71–88%) (Supplementary Table 1). This is reasonable as aligning the reads to their corresponsive genomes probably have allowed detection of genes specific to each line, even though we only focused on 14,206 genes that were annotated on all three reference genomes. The reason was that we wanted to compare all three transcriptomes and that some genes were not annotated across all three reference genomes. Focusing on this set of 14,206 genes did leave out a portion of genes that could be differentially expressed, but the majority (76%) of the genes were included as compared to the 18,503 genes when all three meiocyte transcriptomes were aligned to the same B73 v5 reference [43],Supplementary Fig. 3A; Supplementary Tables 2 and 5). To compensate for this limitation, we aligned all three meiocyte transcriptomes to each of the same B73 v5 reference, Mo17 v1 reference, or CML228 v1 reference (Supplementary Tables 5, 6, and 7; Supplementary Figs. 3, 4, 5, and 6). After all, either approach would have limitations and aligning all three meiocytes to the same reference has relatively similar results to aligning to their corresponding reference, resulting in identification of enriched membrane genes for example. If GO enrichment tools can be improved to take in gene names of Mo17 and CML228 as inputs (currently the ShinyGO 0.76.2 application we are using only takes in B73 gene names), those genes specific to each of the genomes should be investigated. This should give the most complete differential gene expression lanscape that was partially missing by our current approach.

The percentage of reads aligned has been improved from the previous publication, 78–89% (aligning B73 meiocytes to B73 reference v2; [34–36], to the current study, 86–92% (aligning to the B73 reference v5), which makes sense as the reference genome becomes better annotated. The relatively lower percentage of reads mapped for CML228 could be due to that the CML228 v1 reference genome is less complete than the other two reference genomes. Based on assembly metrics, CML228 v1 reference genome has a lower contig N50 than B73 v5 (9.6 vs 52.4 Mb) and a lower number of pan genes than the temperate reference genomes [21]. These results highlight the challenges of doing read alignments of next-generation sequencing data involving multiple diverse genotypes.

Out of the 51 differentially expressed genes enriched in “cell cycle” GO category, 18 genes did not have a GO term. Dukowic-Schulze, Harris, et al. [20] found that maize had much fewer GO terms annotated than Arabidopsis (194 vs. 51 with Revigo, 64 vs. 0 with AgriGO) even though maize has more genes (~ 39,000 in B73 v5) than Arabidopsis (~ 27,000 in TAIR10). Additionally, many GO terms for reproduction, including meiosis and flower organ development, were found to be abundant in analysis of Arabidopsis genes but absent in analysis of maize genes. This suggests that genetics of meiosis and recombination in maize remains to be better studied, including improving reference genome annotation.

## Conclusions

In conclusion, we found that meiotic related genes were mostly conserved among the three maize inbreds except a few DSB- repair/meiotic genes for class I COs and *Zyp1* which limits newly formed class I COs were up-regulated, while *Mus81 homolog 2* for class II COs was down-regulated in CML228. While these observations might be related to the previously observed lower chiasmata number observed in CML228 [15], epigenetic modifications might also play a role. We also found GO categories in membrane, localization, proteolysis, energy processes were up-regulated in CML228 while, cell cycle related processes were down-regulated in CML228. The direction of gene expression of these processes agrees with that previously found in meiotic tissues compared with vegetative tissues. In summary, we used natural maize inbred lines that were from different climatic conditions and have shown their differences in expression landscape in male meiocytes.

## Methods

### Plant materials

CML228 is a tropical maize inbred line of tropical origin, while B73 and Mo17 are both temperate maize lines belonging to different heterotic groups [52]. They were all grown in the same greenhouse facility at University of Minnesota, St. Paul, where the anthers were collected in two batches. The first batch collected contained two replicates of B73, two replicates of Mo17, and one replicate of CML228. A previous publication from our group described the greenhouse conditions used in growing the plants for this first batch, as well as initial gene expression analyses [34–36]. Briefly, the plants were grown in the greenhouse at 16 h of light at 24°C and 8 h of darkness at 22°C in a 2:1 mix of top soil and SunGro LC8. The plants were fertilized with ~ 30 g of Osmocote 14–14-14 slow release fertilizer and afterwards biweekly of ~ 1-2 g Peterson’s 20–20-20 dissolved in water. To be able to complete the comparisons, another two biological replicates of CML228 meiocytes were collected in the second batch. For all inbred lines, one biological replicate consists of a pool of meiocytes from anthers of multiple plants that were grown in a staggered manner over a couple of weeks. Even though the two CML228 replicates were collected some time later, they correlated well with the first replicate from the first batch (Fig. 1).

### Meiocyte isolation and RNA extraction

Meiocytes of CML228, B73, and Mo17 were isolated using a previously established protocol [20, 34, 34, 35, 35, 36, 36, 53]. Briefly, anthers were staged by acetocarmine staining and those containing meiocytes at zygotene stage were squashed on a slide with 1X phosphate buffer saline (PBS) with RNase inhibitor to release the meiocytes. To ensure that meiocytes were at zygotene stage, only upper florets of the mid part of the main tassel branch were collected which develop mostly synchronously. A mouth-controlled glass pipette was then used to collect the meiocytes. Collected meiocytes were stored in -70˚C until RNA extraction. RNA of the new CML228 replicates was extracted using TriZol reagent (Sigma-Aldrich) with the DirectZol RNA kit (Zymo Research) according to the manufacturer’s protocol.

### RNA-seq, differential expression analysis, and Gene Ontology analysis

Two new replicates of CML228 meiocyte RNA were sent to the University of Minnesota Genomics Center for TruSeq RNA-seq library preparation (Illumina) and sequenced using HiSeq 2500 1 × 50 bp run. A total of two libraries were created (one for each biological replicate) and sequenced with at least 40 million reads per library. These two CML228 replicates (NCBI-SRA BioProject PRJNA396253 [SRR14498235 and SRR14498234]), together with the previously generated one replicate of CML228 (PRJNA396253 [SRR5930250] sequenced using HiSeq 2000 1 × 150 bp), and two replicates of each of B73 (PRJNA185817 [SRR650380 and SRR650383]) and Mo17 (PRJNA396254 [SRR5931450 and SRR5931453] [34–36], a total of seven samples, were used for the differential expression analysis.

Differential expression analysis was done in Galaxy [54]. Quality control and removal of adapter sequences were done on all three inbred lines using Trimmomatic with the sliding window trimming with an average across 4 bases and a minimal 25 average quality [55]. STAR was then used to align the good quality sequences of the B73 meiocytes against the B73 maize genome sequence version 5 assembly, Mo17 meiocytes against Mo17 version 1 CAU assembly, and CML228 meiocytes against the CML228 version 1 assembly with 49 bp genomic sequence around annotated junctions [21, 22, 56]. The aligned reads of each sample were sorted using Samtools sort and then quantified using StringTie with default parameters and showing the reference transcripts only [57, 58]. As outputs from StringTie, for each sample, a gene abundance estimate file was produced which contained RPKM values, the number of reads for a gene normalized by gene length in kilobases and sequencing depth in millions, and a gene count file was produced which was used for DESeq2 analysis. Genes with an average RPKM value > 2 across the seven samples aligned to the same reference were kept (all seven samples were aligned to each of the three references in addition to each meiocytes aligning to their corresponsive genome). The resulting gene count files of each sample aligned to their corresponding reference and only genes that were present on all three references were used for the differential expression analysis using DESeq2 [59]. Z-scores, as a way to quantify gene expression, were calculated on the resulting normalized counts for each gene, as ${z}_{i,j}=\frac{{x}_{i,j}-{\overline{x} }_{i}}{{s}_{i}}$, where *z*_*i,j*_ is the Z-score for gene *i* in sample *j*, *x*_*i,j*_ is the normalized count, ${\overline{x} }_{i}$ is the mean normalized count for gene *i*, and *s*_*i*_ is the corresponding standard deviation. Heatmap2 was used to plot the Z-scores of the most differentially expressed genes (R gplots package v3.0.1 and rcolorbrewer v1.1_2). Genes with a log_2_ fold change ≥ 1 (upregulated) or ≤ -1 (downregulated) and a FDR ≤ 0.01 (after Benjamini–Hochberg correction) were used to define differentially expressed genes.

To identify the nature of these differentially expressed genes, a Gene Ontology (GO) enrichment analysis of genes that were up- and down-regulated in CML228 compared with B73 or Mo17 was conducted using ShinyGO 0.76.2 at a false discovery rate (FDR) cutoff of 0.05 showing the top 30 GO pathways [23]. For up-regulated genes, down-regulated genes in CML228, and genes in a few selected GO categories, the enriched top 10 KEGG pathways were further visualized and rendered by Pathview [23–27].

### Gene expression validation

Gene expression of a differentially expressed gene, *ZmZyp1*, was validated using quantitative real-time PCR (qPCR) with ubiquitin (GRMZM2G419891, V3 Maize Annotation) as a reference gene. Ubiquitin as a reference gene has been shown to have stable expression in qRT-PCR studies in maize [60, 61]. RNA of each of CML228, B73, and Mo17 maize line was extracted from zygotene anthers of three plants to represent three biological replicates. RNA concentrations were adjusted for each sample and then reverse-transcribed using Superscript III First Strand Synthesis Mix (Invitrogen). Real-time PCR was done using BioRad iQ SYBR Green Supermix in a BioRad CFX96 real time PCR machine. Analysis was done using the integrated BioRad CFX Manager software. The PCR primers used were the following: *ZmZyp1*_F – CGACGAGCACCCACCAG, *ZmZyp1*_R – TGCTCCTTGACTAATTTCTCTGCT, Z*mUbi*_F – CGCACCCTAGCAGACTACAA, and *ZmUbi*_R – TACGCACACACAACACAACC. Relative expression of *Zyp1* was compared among CML228, B73 and Mo17 using Tukey’s HSD test with an ANOVA linear model fitting the maize line and replication as random effects.

## Supplementary Information

**Additional file 1: Supplementary Figure 1.** Genes expressed with RPKM>2 in each of the B73, Mo17, and CML228 meiocytes aligned to each of their corresponding genomes. The 14206 genes were used for subsequent analysis. **Supplemental Figure 2. **Clustering of meiocyte transcriptomes of B73, Mo17, and CML228 maize inbred lines aligned to their corresponding reference genomes. (**A**) Heatmap showing sample-to-sample distance matrix. (**B**) principal component analysis. Outputs of DESeq2. **Supplementary Figure 3. **Z-scores showing expression profiles of the differentially expressed genes of all meiocyte samples of maize inbred B73 (SRR650383 and SRR650380), Mo17 (SRR5931453 and SRR5931450), and CML228 (SRR5930250, SRR14498235, and SRR14498234) aligned to (**A**) the B73 v5 reference (4199 genes), (**B**) Mo17 v1 reference (3251 genes), and** (C) **CML228 v1 reference (3536 genes). **Supplementary Figure 4.** Top 30 Gene Ontology (GO) terms associated with (**A**) the 2441 up-regulated genes and (**B**) the 1758 down-regulated genes in CML228 meiocytes compared to B73, Mo17 meiocytes aligned to the B73 v5 reference. **Supplementary Figure 5.** Top 30 Gene Ontology (GO) terms associated with (**A**) the 1965 up-regulated genes and (**B**) the 1286 down-regulated genes in CML228 meiocytes compared to B73, Mo17 meiocytes aligned to the Mo17 v1 reference. **Supplementary Figure 6. **Top 30 Gene Ontology (GO) terms associated with (**A**) the 2218 up-regulated genes and (**B**) the 1318 down-regulated genes in CML228 meiocytes compared to B73, Mo17 meiocytes aligned to the CML228 v1 reference. **Supplementary Figure 7. **qPCR validation of ZmZyp1 relative expression. **Supplementary Figure 8. **Top KEGG pathways for cell cycle genes that were down-regulated in the tropical maize inbred CML228 found through the gene ontology enrichment analysis (Ge, Jung, and Yao 2020; Kanehisa 2019; Kanehisa et al. 2021; Kanehisa and Goto 2000; Luo and Brouwer 2013).

**Additional file 2: Supplementary Table 1. **Percent of aligned reads for the B73, Mo17, and CML228 meiocytes to B73 v5 reference, Mo17 v1 reference, and CML228 v1 reference. **Supplementary Table 2.** Genes that had a reads per kilobase million (RPKM) value greater than 2 and were present in B73, Mo17, and CML228 meiocytes aligned to each of their corresponsive genomes (the B73 v5 reference, Mo17 v1 reference, and the CML228 v1 reference). Values shown for each sample were Z-scores, indicating levels of gene expression. **Supplementary Table 3.** Common meiosis-related genes detected in temperate maize lines B73 and Mo17 and the tropical maize line CML228 male meiocytes. Values shown for each sample were Z-scores, indicating levels of gene expression. **Supplementary Table 4.** Significantly differentially expressed genes in "cell cycle" gene ontology category: their expression levels (shown in Z-scores) and functions. **Supplementary Table 5.** Genes that had a reads per kilobase million (RPKM) value greater than 2 of B73, Mo17, and CML228 meiocytes aligned to the B73 v5 reference genome. Values shown for each sample were Z-scores, indicating levels of gene expression. **Supplementary Table 6.** Genes that had a reads per kilobase million (RPKM) value greater than 2 of B73, Mo17, and CML228 meiocytes aligned to the Mo17 v1 reference genome. Values shown for each sample were Z-scores, indicating levels of gene expression.** Supplementary Table 7.** Genes that had a reads per kilobase million (RPKM) value greater than 2 of B73, Mo17, and CML228 meiocytes aligned to the CML228 v1 reference genome. Values shown for each sample were Z-scores, indicating levels of gene expression.

## Data Availability

The datasets generated for this study for two replicates of CML228 are deposited at the NCBI-SRA under PRJNA396253 (SRR14498235 and SRR14498234). The data generated earlier for one replicate of CML228 was deposited at PRJNA396253 (SRR5930650). Datasets from previous studies for B73 and Mo17 can be found at the NCBI-SRA under PRJNA185817 (SRR650380 and SRR650383) and PRJNA396254 (SRR5931450 and SRR5931453), respectively.

## Abbreviations

DSB: Double strand break
CO: Crossover
CDK: Cyclin-dependent kinase
RPKM: Reads per kilobase per million
GTP: Guanosine triphosphate
GO: Gene Ontology
KEGG: Kyoto Encyclopedia of Genes and Genomes
qPCR: quantitative PCR/real-time PCR
ER: Endoplasmic reticulum
Mpk3: mitogen-activated protein kinase 3
ATM: Ataxia telangiectasia mutated
ATR: ATM- and RAD3-related
H3K4me3: Trimethylation of lysine 4 of the H3 histone
RPA: Replication protein A
RFC: Replication factor C
dHJ: Double Holliday junction
