# Supplementary material for: Comparison of meiotic transcriptomes of three maize inbreds with different origins reveals differences in cell cycle and recombination
Source: BMC Genomics. 2022 Oct 12;23:702. doi: 10.1186/s12864-022-08922-w (PMC9554999; doi:10.1186/s12864-022-08922-w)
Supplement: Supplementary file 1 — Additional file 1: Supplementary Figure 1. Genes expressed with RPKM>2 in each of the B73, Mo17, and CML228 meiocytes aligned to each of their corresponding genomes. The 14206 genes were used for subsequent analysis. Supplemental Figure 2. Clustering of meiocyte transcriptomes of B73, Mo17, and CML228 maize inbred lines aligned to their corresponding reference genomes. (A) Heatmap showing sample-to-sample distance matrix. (B) principal component analysis. Outputs of DESeq2. Supplementary Figure 3. Z-scores showing expression profiles of the differentially expressed genes of all meiocyte samples of maize inbred B73 (SRR650383 and SRR650380), Mo17 (SRR5931453 and SRR5931450), and CML228 (SRR5930250, SRR14498235, and SRR14498234) aligned to (A) the B73 v5 reference (4199 genes), (B) Mo17 v1 reference (3251 genes), and (C) CML228 v1 reference (3536 genes). Supplementary Figure 4. Top 30 Gene Ontology (GO) terms associated with (A) the 2441 up-regulated genes and (B) the 1758 down-regulated genes in CML228 meiocytes compared to B73, Mo17 meiocytes aligned to the B73 v5 reference. Supplementary Figure 5. Top 30 Gene Ontology (GO) terms associated with (A) the 1965 up-regulated genes and (B) the 1286 down-regulated genes in CML228 meiocytes compared to B73, Mo17 meiocytes aligned to the Mo17 v1 reference. Supplementary Figure 6. Top 30 Gene Ontology (GO) terms associated with (A) the 2218 up-regulated genes and (B) the 1318 down-regulated genes in CML228 meiocytes compared to B73, Mo17 meiocytes aligned to the CML228 v1 reference. Supplementary Figure 7. qPCR validation of ZmZyp1 relative expression. Supplementary Figure 8. Top KEGG pathways for cell cycle genes that were down-regulated in the tropical maize inbred CML228 found through the gene ontology enrichment analysis (Ge, Jung, and Yao 2020; Kanehisa 2019; Kanehisa et al. 2021; Kanehisa and Goto 2000; Luo and Brouwer 2013). [file 12864_2022_8922_MOESM1_ESM.docx]

Supplementary Figures


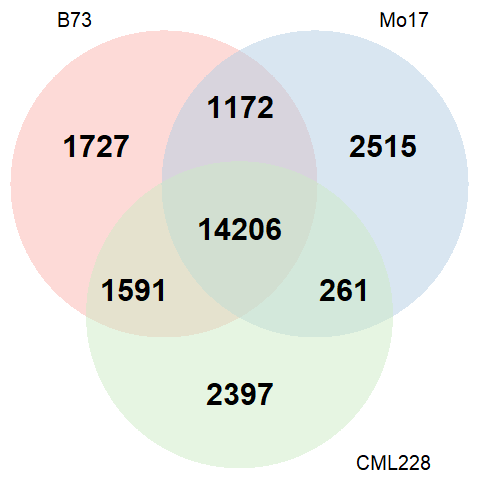


**Supplementary Figure 1. Genes expressed with RPKM>2 in each of the B73, Mo17, and CML228 meiocytes aligned to each of their corresponding genomes.** The 14206 genes were used for subsequent analysis.


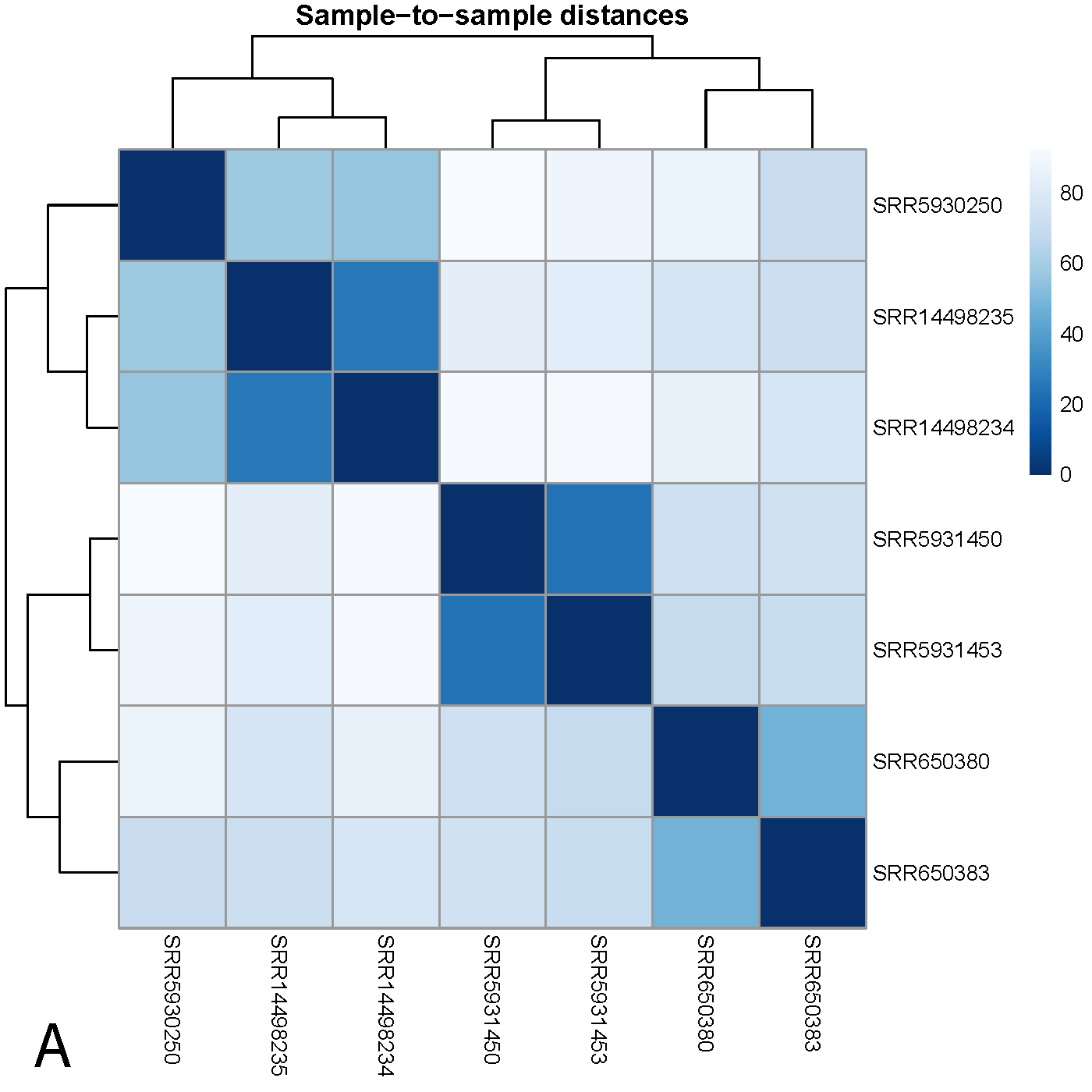


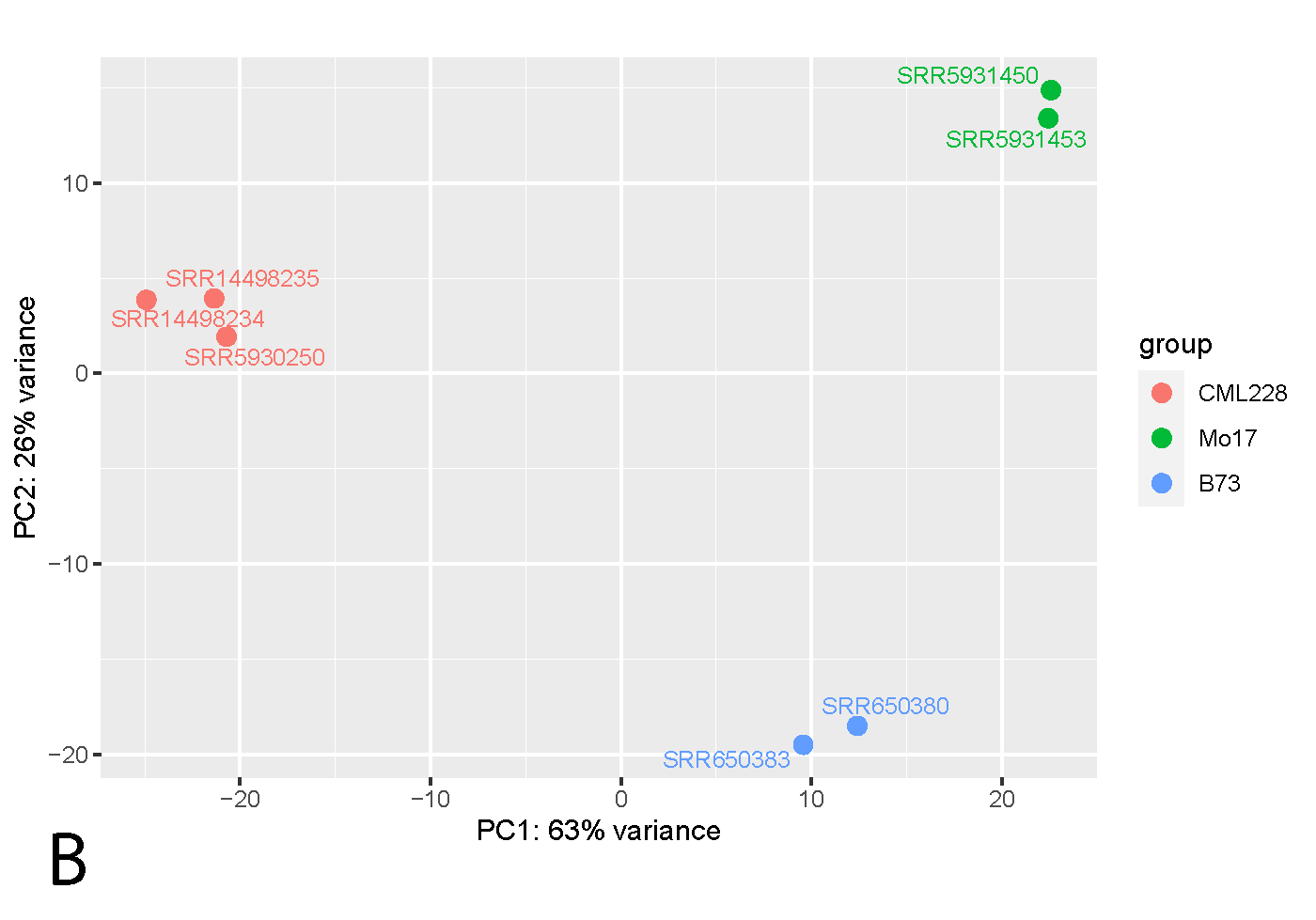


**Supplemental Figure 2. Clustering of meiocyte transcriptomes of B73, Mo17, and CML228 maize inbred lines aligned to their corresponding reference genomes.** (**A**) Heatmap showing sample-to-sample distance matrix. (**B**) principal component analysis. Outputs of DESeq2.


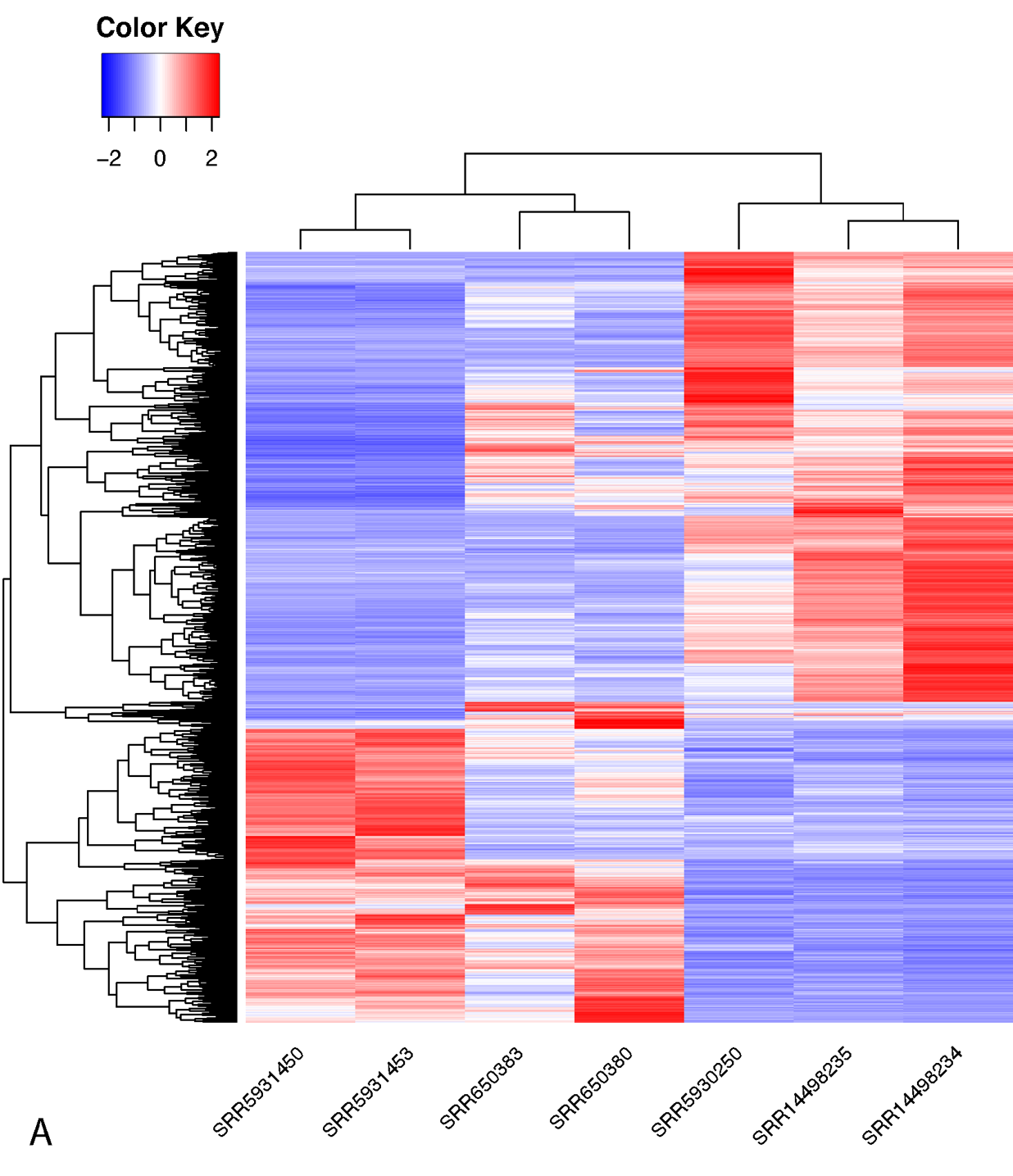


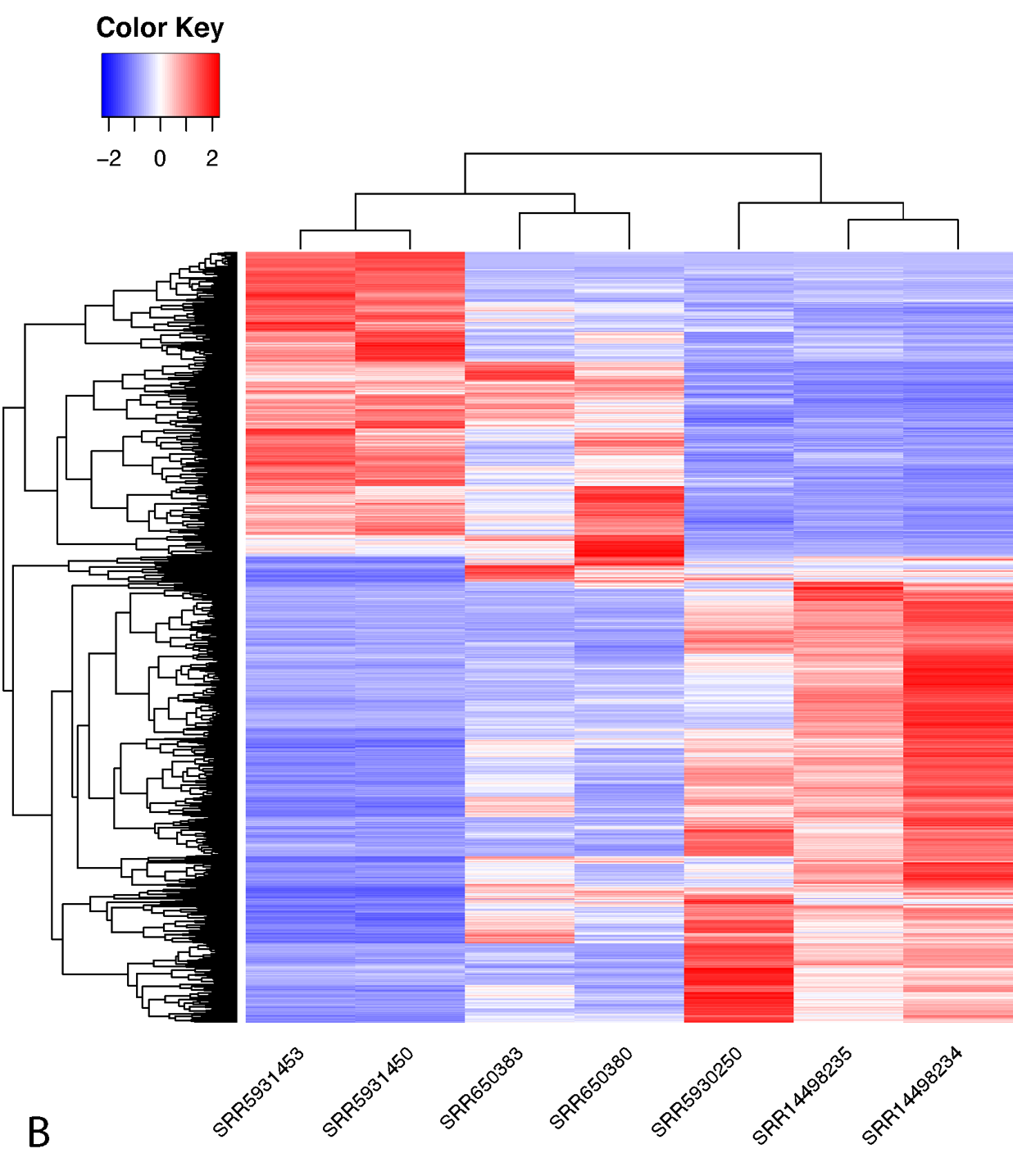


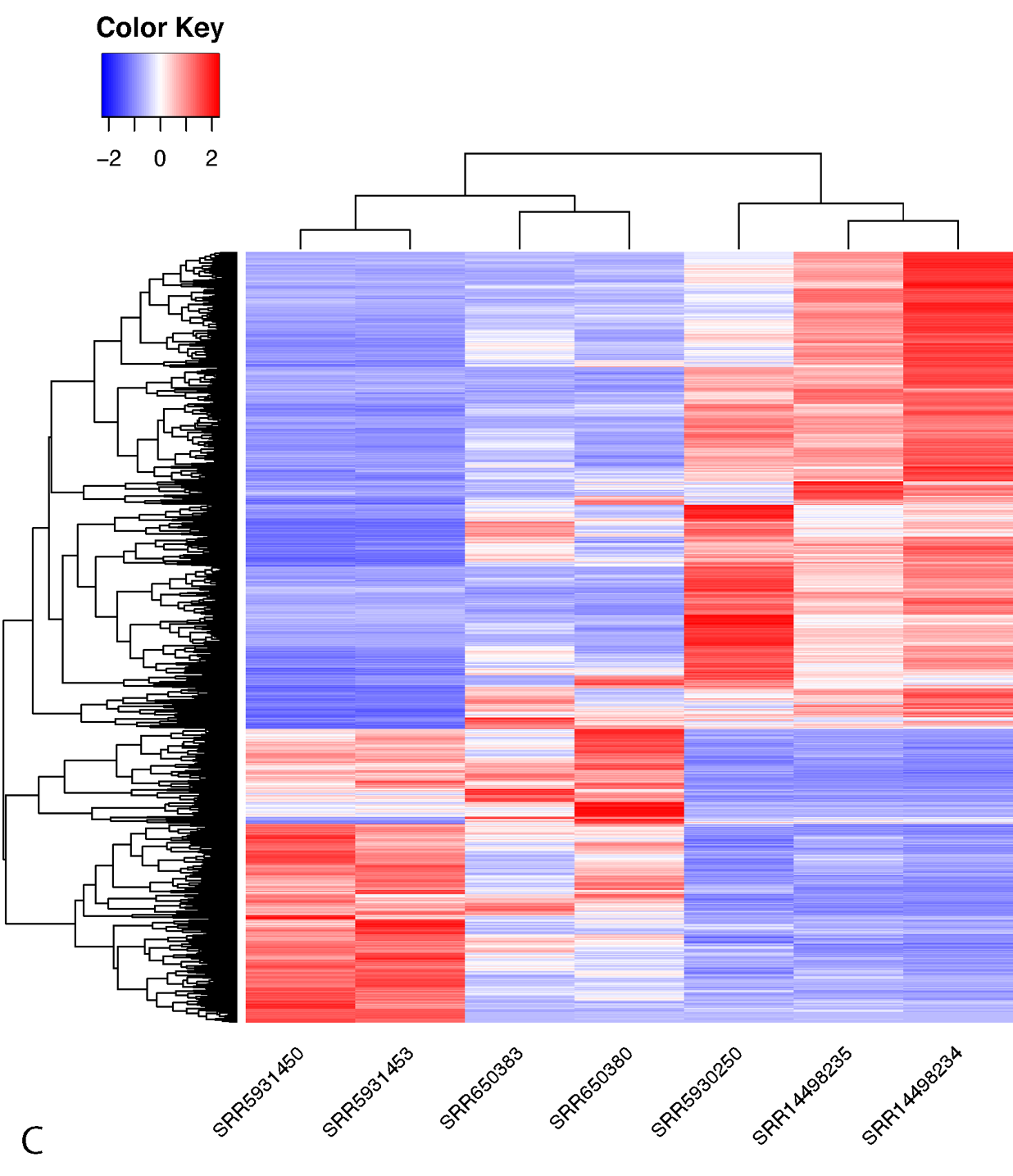


**Supplementary Figure 3. Z-scores showing expression profiles of the differentially expressed genes of all meiocyte samples of maize inbred B73** (SRR650383 and SRR650380), **Mo17** (SRR5931453 and SRR5931450), **and CML228** (SRR5930250, SRR14498235, and SRR14498234) **aligned to (A) the B73 v5 reference (4199 genes), (B) Mo17 v1 reference (3251 genes), and (C) CML228 v1 reference (3536 genes.**

**
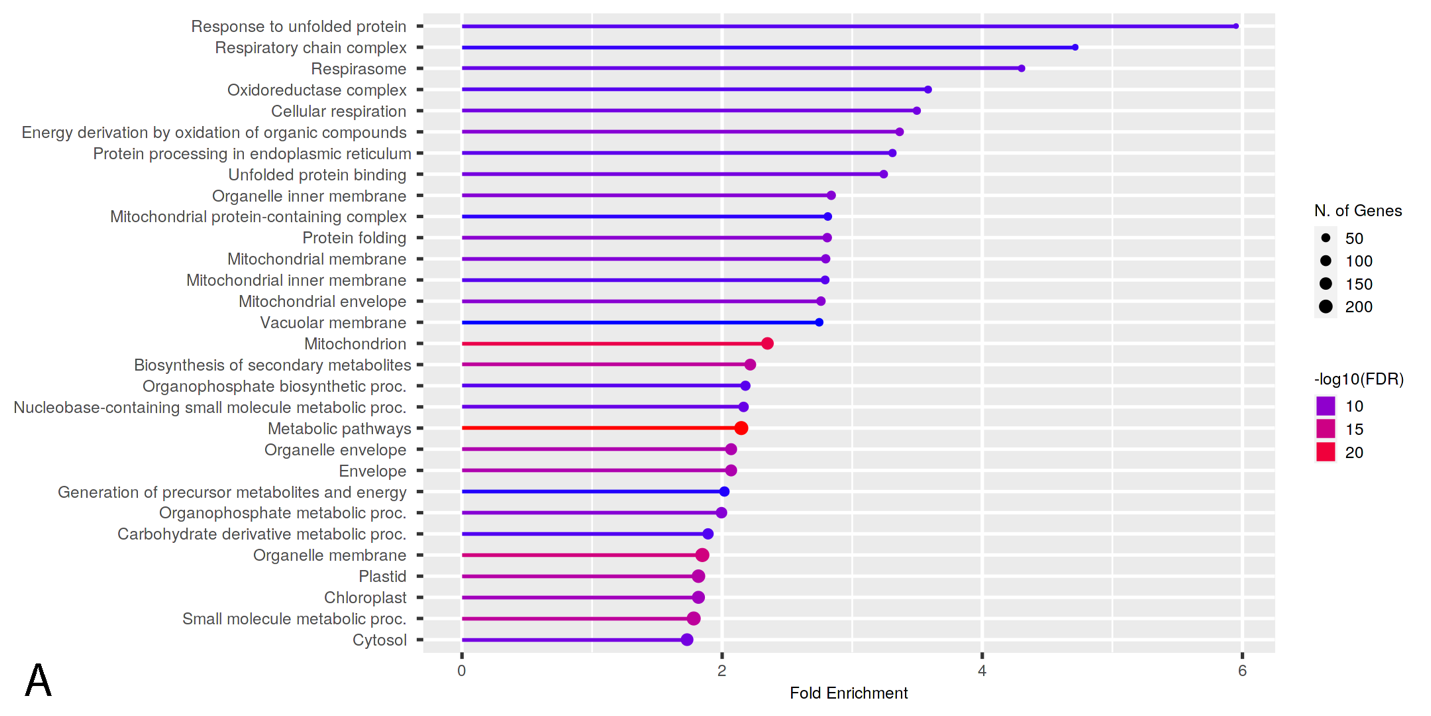
**

**
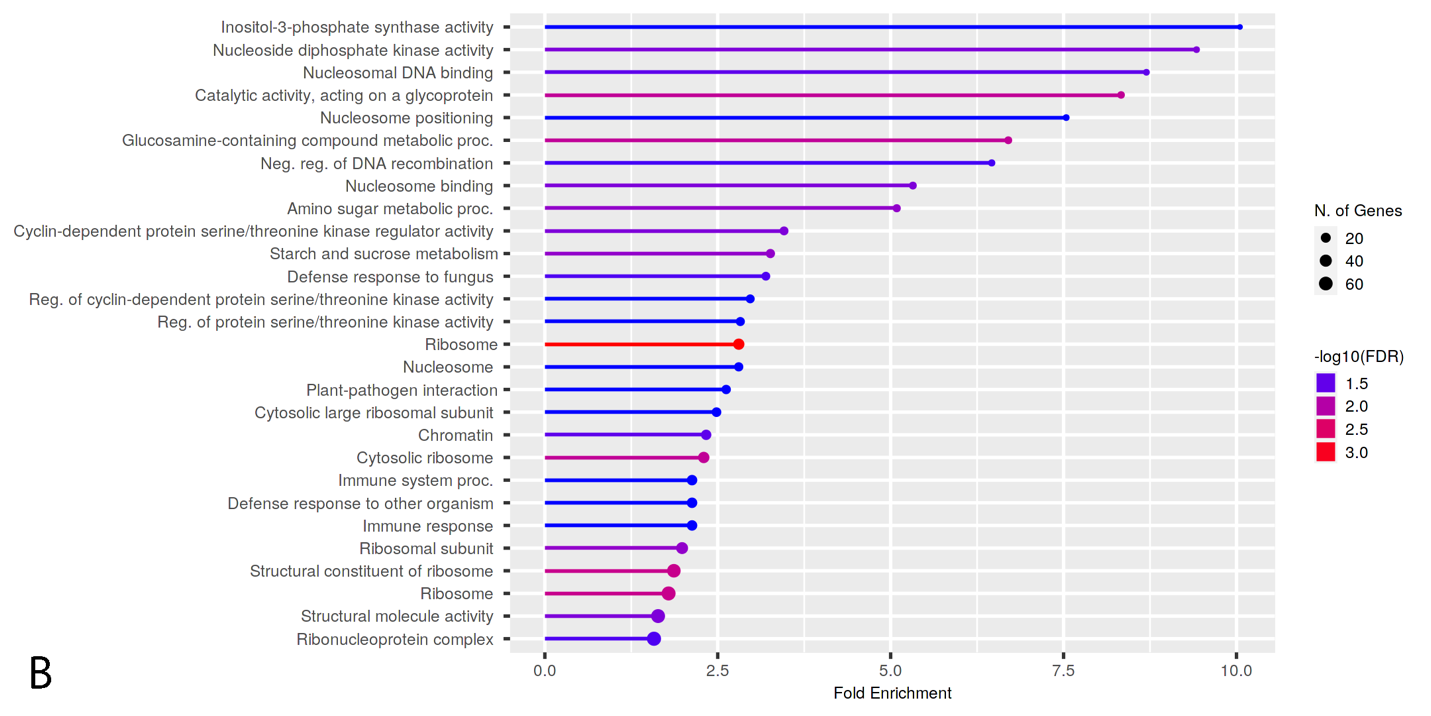
**

**Supplementary Figure 4.**  **Top 30 Gene Ontology (GO) terms associated with (A) the 2441 up-regulated genes and (B) the 1758 down-regulated genes in CML228 meiocytes compared to B73, Mo17 meiocytes aligned to the B73 v5 reference.**

**
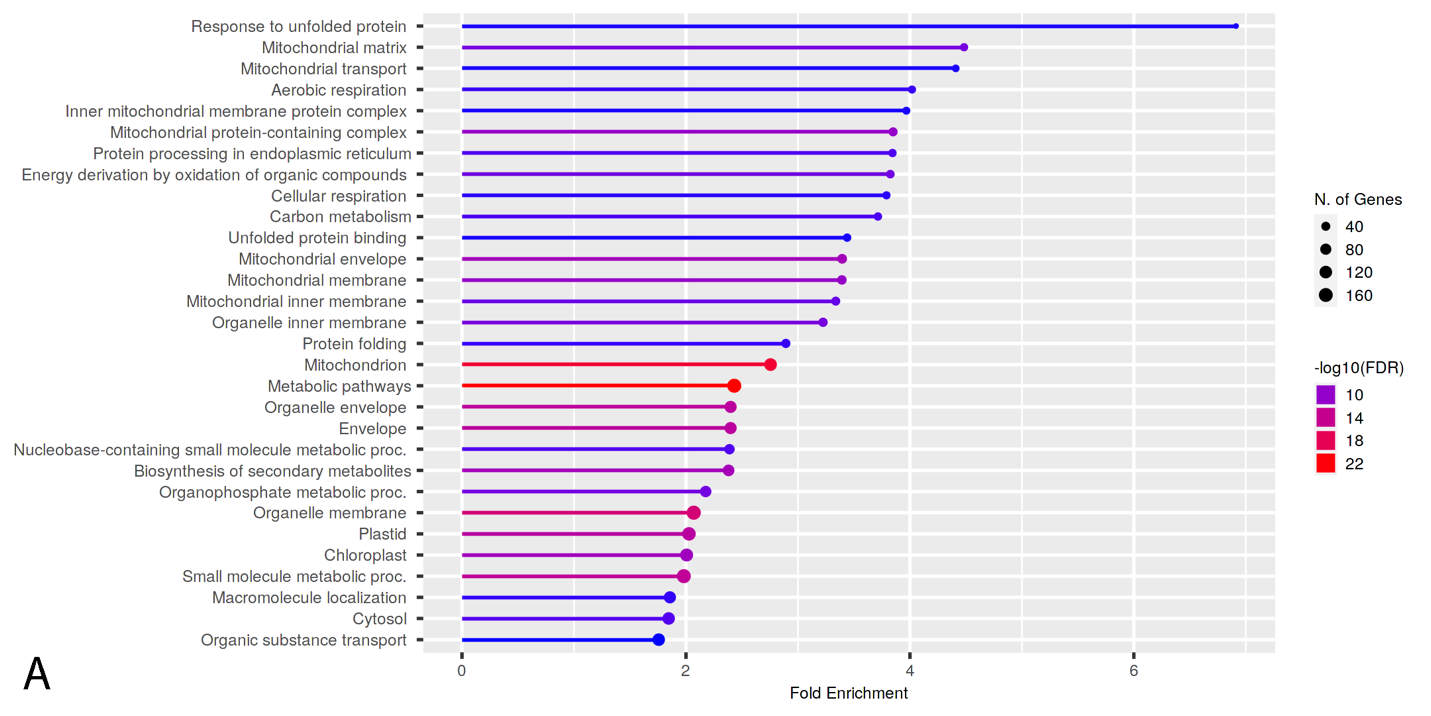
**

**
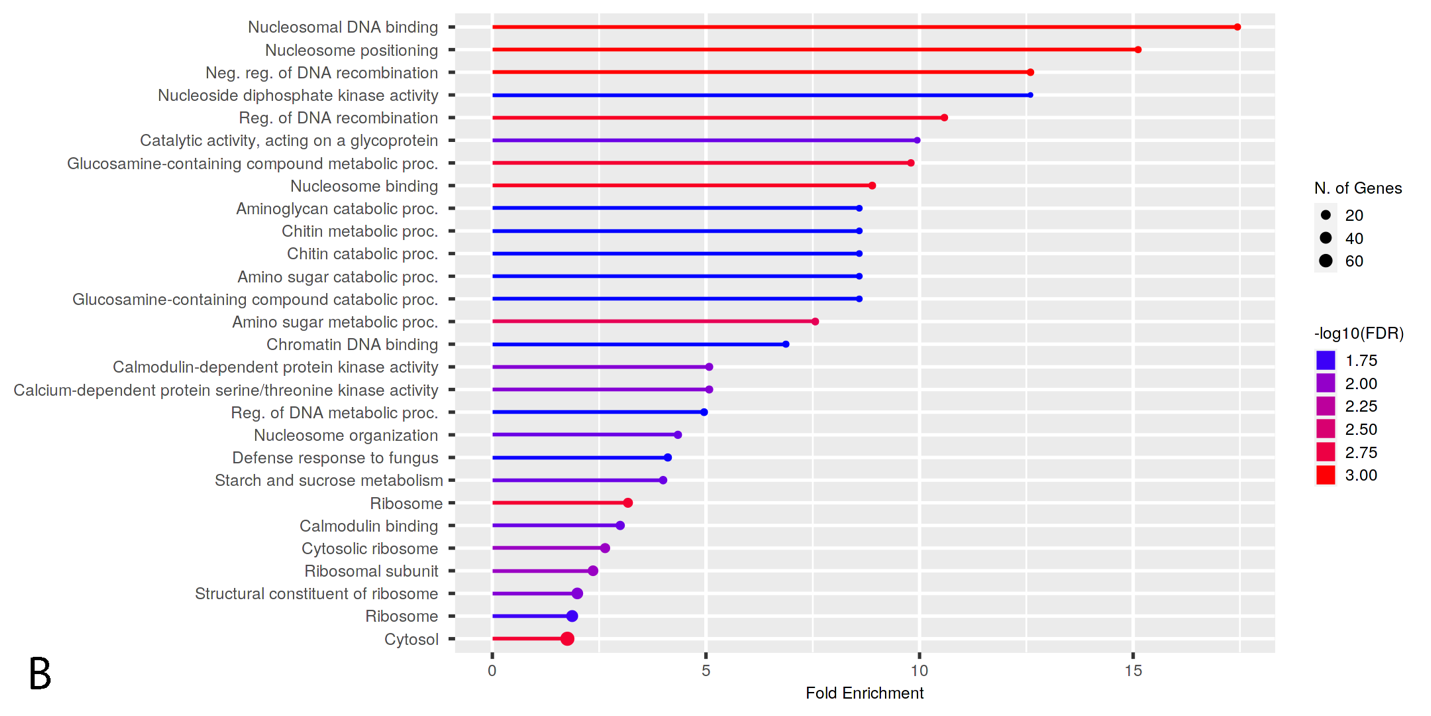
**

**Supplementary Figure 5.**  **Top 30 Gene Ontology (GO) terms associated with (A) the 1965 up-regulated genes and (B) the 1286 down-regulated genes in CML228 meiocytes compared to B73, Mo17 meiocytes aligned to the Mo17 v1 reference.**

**
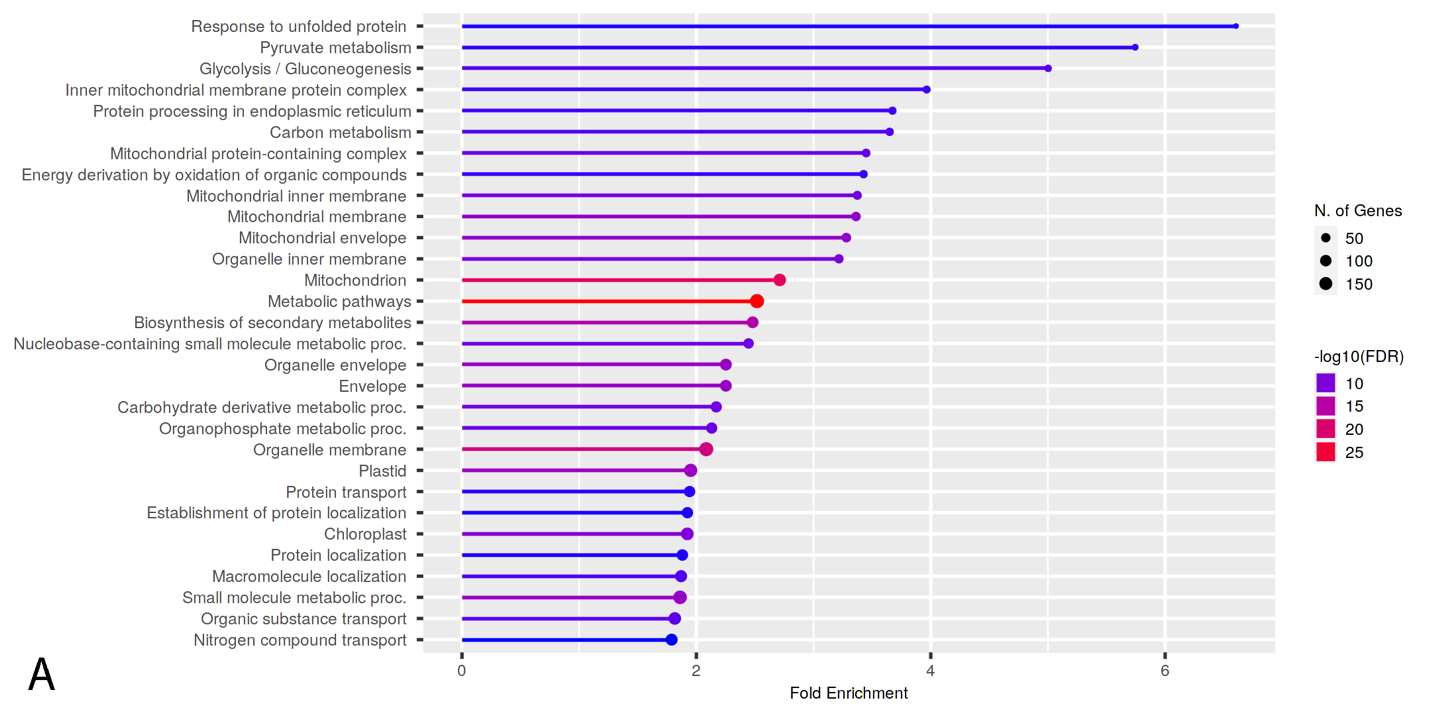
**

**
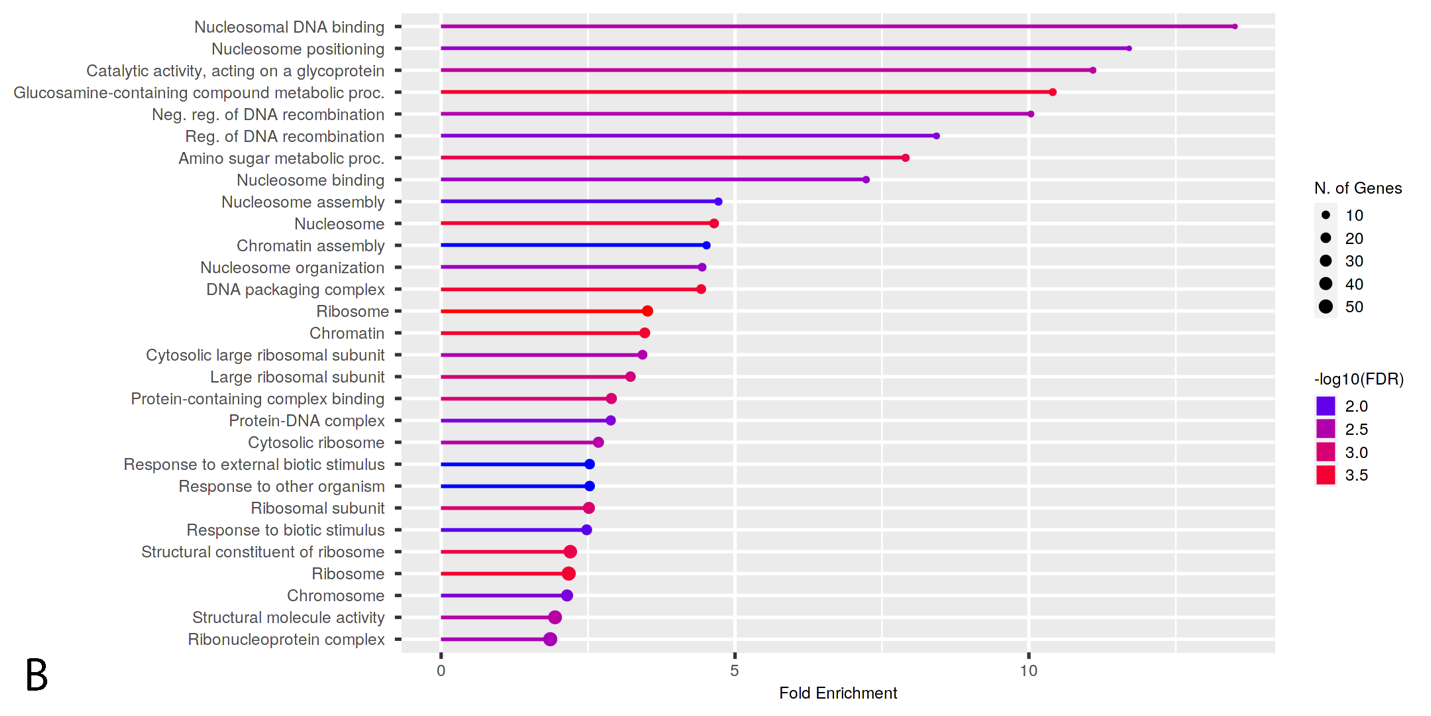
**

**Supplementary Figure 6.**  **Top 30 Gene Ontology (GO) terms associated with (A) the 2218 up-regulated genes and (B) the 1318 down-regulated genes in CML228 meiocytes compared to B73, Mo17 meiocytes aligned to the CML228 v1 reference.**

**
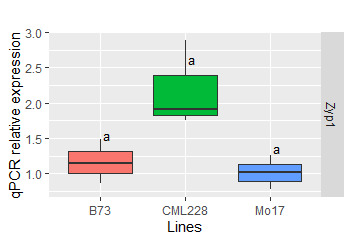
**

**Supplementary Figure 7. qPCR validation of ZmZyp1 relative expression.**

**
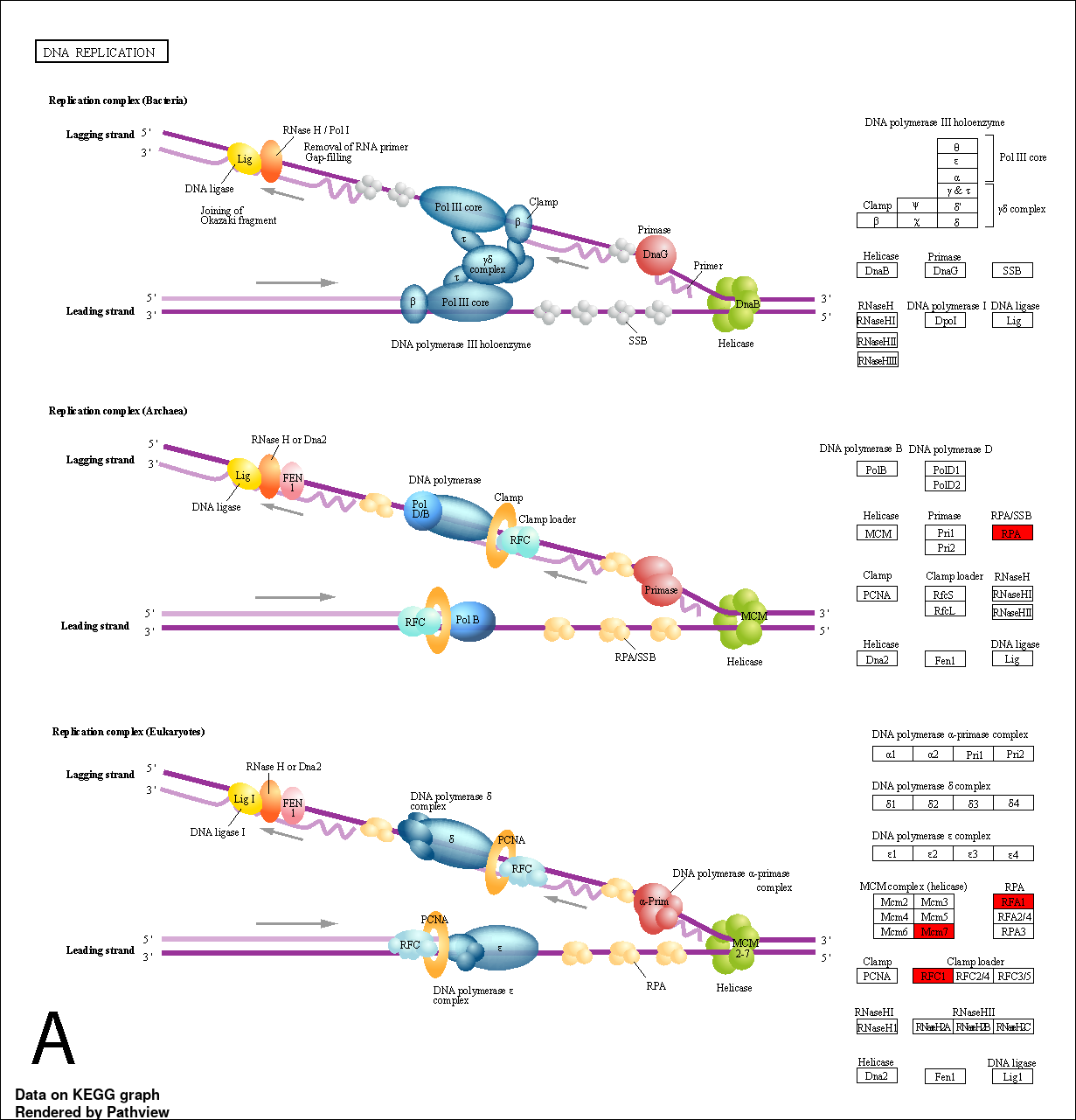
**

**

**

**
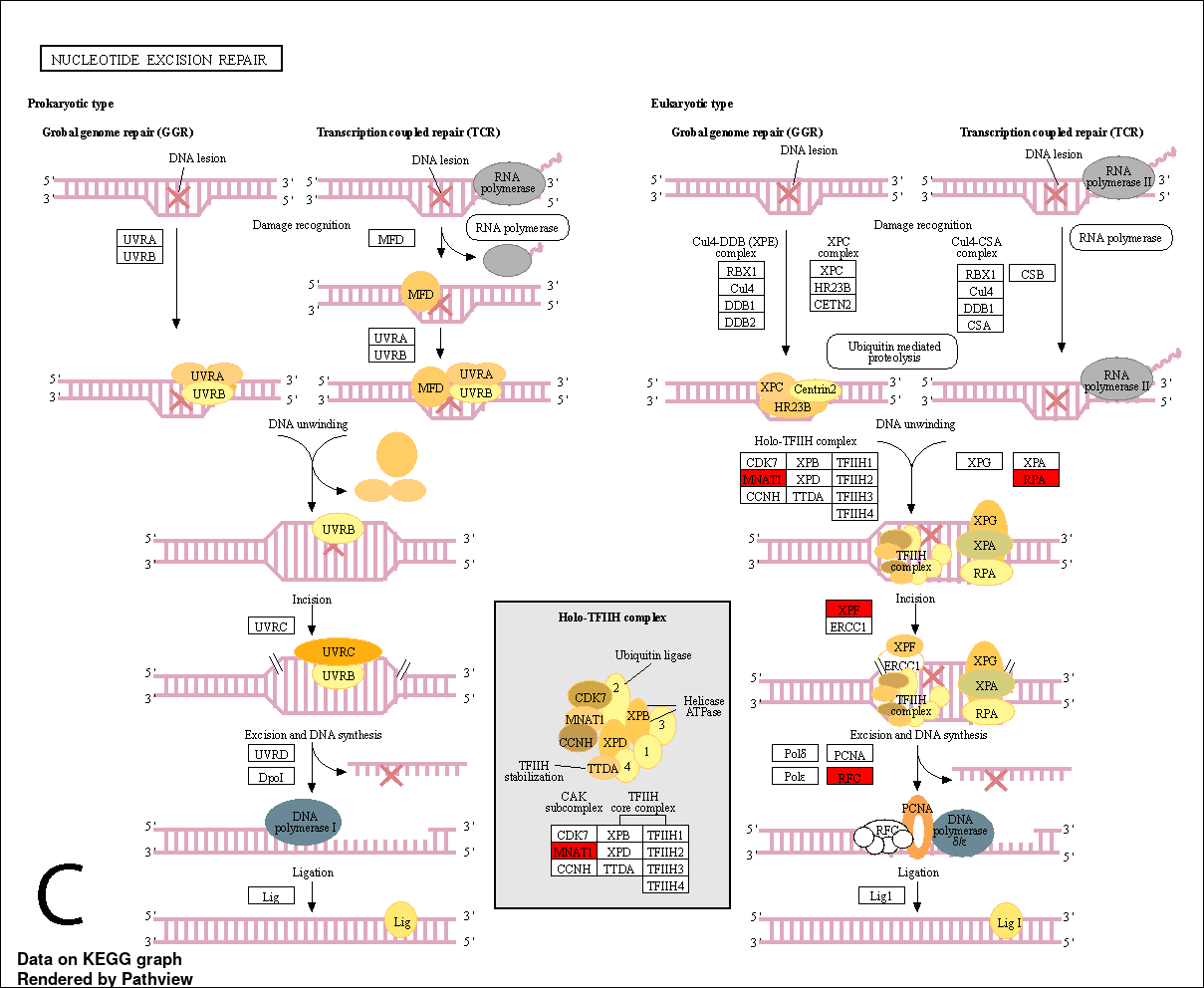
**

**Supplementary Figure 8. Top KEGG pathways for cell cycle genes that were down-regulated in the tropical maize inbred CML228 found through the gene ontology enrichment analysis (Ge, Jung, and Yao 2020; Kanehisa 2019; Kanehisa et al. 2021; Kanehisa and Goto 2000; Luo and Brouwer 2013).**
